# Quantitative trait locus (QTL) analysis and fine-mapping for *Fusarium oxysporum* disease resistance in *Raphanus sativus* using GRAS-Di technology

**DOI:** 10.1270/jsbbs.23032

**Published:** 2023-11-01

**Authors:** Chukwunonso Sylvanus Austin Ezeah, Juichi Shimazu, Takahiro Kawanabe, Motoki Shimizu, Shinichi Kawashima, Makoto Kaji, Charles Onyemaechi Ezinma, Md Nuruzzaman, Nami Minato, Eigo Fukai, Keiichi Okazaki

**Affiliations:** 1 Laboratory of Plant breeding, Graduate School of Science and Technology, Niigata University, 2-8050 Ikarashi, Nishi-ku, Niigata 950-2181, Japan; 2 Nanto Seed Co., Ltd., Kashihara, Nara 634-0077, Japan; 3 School of Agriculture, Tokai University, Kumamoto 862-0970, Japan; 4 Iwate Biotechnology Research Center, Kitakami, Iwate 024-0003, Japan; 5 Watanabe Seed Co., Ltd., Miyagi 987-0003, Japan; 6 Federal Department of Agriculture, Federal Ministry of Agriculture and Rural Development, Abuja, FCT, Nigeria; 7 Department of Genetics and Plant Breeding, Bangladesh Agricultural University, Mymensingh, Bangladesh

**Keywords:** disease, fine-map, fusarium, GRAS-Di, QTL, radish, resistance

## Abstract

Fusarium wilt is a significant disease in radish, but the genetic mechanisms controlling yellows resistance (YR) are not well understood. This study aimed to identify YR-QTLs and to fine-map one of them using F_2:3_ populations developed from resistant and susceptible radish parents. In this study, two high-density genetic maps each containing shared co-dominant markers and either female or male dominant markers that spanned 988.6 and 1127.5 cM with average marker densities of 1.40 and 1.53 cM, respectively, were generated using Genotyping by Random Amplicon Sequencing-Direct (GRAS-Di) technology. We identified two YR-QTLs on chromosome R2 and R7, and designated the latter as *ForRs1* as the major QTL. Fine mapping narrowed down the *ForRs1* locus to a 195 kb region. Among the 16 predicted genes in the delimited region, 4 genes including two receptor-like protein and -kinase genes (RLP/RLK) were identified as prime candidates for *ForRs1* based on the nucleotide sequence comparisons between the parents and their predicted functions. This study is the first to use a GRAS-Di for genetic map construction of cruciferous crops and fine map the YR-QTL on the R7 chromosome of radish. These findings will provide groundbreaking insights into radish YR breeding and understanding the genetics of YR mechanism.

## Introduction

Radish (*Raphanus sativus* L., 2n = 2x = 18), a member of the family of *Brassicaceae*, is an important root vegetable that is very popular worldwide. It is consumed in a variety of ways such as root vegetable, salad, leafy vegetable, etc., and it is an important source of minerals and vitamins in the human diet. Radish production is seriously threatened by Fusarium wilt (FW), a disease caused by the soil-borne fungus *Fusarium oxysporum* f. sp. *raphani* (FOR). The pathogen attacks radishes by penetrating through mechanical damage on the root surface, extends through the vascular tissues, and spreads throughout the internal tissues, thereby causing stunted growth, wilting and yellowing of leaves, which finally results in the plant’s death (Garibaldi *et al.* 2006, Gordon 2017). Radish yellows was first discovered in California, the United States, in 1934 (Kendrick and Snyder 1936), and then spread to Wisconsin (Pound 1953), and Washington (Du Toit and Pelter 2003). In Japan, it was discovered in Wakayama Prefecture in 1952, and then spread throughout Japan (Hida and Ashizawa 1985). Radish has a large production area worldwide, which makes it difficult to control FW with agrochemicals (Hida and Ashizawa 1985), and conventional farming methods are not very effective because the spores remain in the soil for a long period of time without a host (Gordon 2017). Therefore, the best method for its control is the use of Fusarium yellows resistance (YR) cultivars (Ma *et al.* 2021, Pu *et al.* 2012).

Over the past decade, Quantitative Trait Loci (QTLs) and fine mapping analyses had been conducted in crops of the family *Brassicaceae* for identification of YR genes and elucidating resistance mechanisms to FW. In QTL and segregation studies on *Brassica oleracea*, Pu *et al.* (2012) identified a YR-QTL, *FocBo1*, as a single dominant gene that confers resistance to FW caused by *F. oxysporum* f. sp. *conglutinans*, and was mapped to the linkage group, C7. Lv *et al.* (2013) identified a *FOC_1_* gene on cabbage chromosome C6 as the YR gene to *F. oxysporum* f. sp. *conglutinans*, based on phenotypic assay of 160 double haploid (DH) lines, derived from the F_1_ of a cross between the resistant and susceptible cabbage parents. In *B. rapa*, Bra012688 and Bra012689 genes were identified as candidate genes for YR in Chinese cabbage (*B.* *rapa* var. *pekinensis*) by Shimizu *et al.* (2014) through differential gene expression analysis, using RNA sequencing. Thereafter, in *B. oleracea*, the YR gene was map-based cloned as an orthologous gene of Bra012688 (Lv *et al.* 2014, Shimizu *et al.* 2015).

In radish, several studies have identified YR-QTLs in different regions of the genome. Kaneko *et al.* (2007) discovered a YR-QTL on R7, which was subsequently identified as corresponding to the long arm of chromosome 1 in *Arabidopsis thaliana* by Shirasawa *et al.* (2011). Yu *et al.* (2013) identified 8 QTLs (*qFW1*-*qFW8*) responsible for YR, and were distributed on 5 LGs (2, 3, 4, 6 and 7). Of these loci, 3 (*qFW3*, *qFW4* and *qFW8*) were constantly detected in three independent tests over 2 years. Since *qFW4*, mapped on LG3 (chromosome R5), accounted for most of the phenotypic variations (14.63%), the locus was recognized as a major QTL and designated as *Fusarium wilt Resistance locus 1* (*Fwr1*) (Yu *et al.* 2013). In the later study, Yu *et al.* (2020) fine-mapped the *Fwr1* to a region of 139.8 kb between 2 DNA markers FM82 and FM87 and identified ORF4 as a YR candidate gene that encodes a serine/arginine-rich protein kinase. In another QTL analysis of radish, Ma *et al.* (2021) detected 2 YR-QTLs, *FoRsR7.1* and *FoRsR9.3* on R7 and R9 chromosomes, respectively, and further argued five putative resistance-related genes in the region of *FoRsR7.1*. Lee *et al.* (2021) reported that the genome-wide association study (GWAS) analysis using 225 radish accessions identified 44 single nucleotide polymorphisms (SNPs) and twenty candidate genes associated with YR. They also did QTL analysis using an F_2_ population derived from a YR line and a susceptible line, and consequently, four QTLs were identified, one of which collocated with the SNP on R7 detected in the GWAS study.

Genetic maps constructed to identify YR-QTLs have been laborious so far, but recent progress of the next generation sequencing (NGS) technology has made it possible to generate linkage maps quickly and efficiently. Genotyping by Random Amplicon Sequencing-Direct (GRAS-Di), one of the Genotyping-by-Sequencing technologies, is a recently developed technique that uses random primers to amplify amplicons to be sequenced (Enoki 2019, Enoki and Takeuchi 2018, Miki *et al.* 2020). This method can be applied to thousands of samples by using primer sets containing different index sequences (Miki *et al.* 2020). Use of GRAS-Di maps has been applied in several cultivars because of the high reproducibility of this method and the very low percentage of missing values (Fekih *et al.* 2023, Miki *et al.* 2020, Moriya *et al.* 2021, Takeshima *et al.* 2022, Umeda *et al.* 2021). However, there are no reports of using this method for linkage mapping in cruciferous crops.

As mentioned above, one of the major YR-QTLs in radishes have been identified on the R7 chromosome (Kaneko *et al.* 2007, Lee *et al.* 2021, Ma *et al.* 2021), and several candidate genes have been proposed based on the co-localization of the identified QTLs. However, it is not known whether the QTLs detected in these different research groups are same or different from each other because of the lack of detailed molecular information of each locus, such as sequence information of the DNA markers that were used to define the loci. Regarding this, in this study, we tried to identify the QTLs responsible for YR in radish using GRAS-Di and candidate genes in *ForRs1*, one of the 2 QTLs detected, then the results were compared to those of previous studies to establish solid basis for YR breeding in radish.

## Materials and Methods

### Plant materials and crossing scheme

F_1_ plants were produced from a cross between the parents, YR RK15-1 and susceptible AKM radish inbred lines (Supplemental Fig. 1). The parental inbred lines were supplied by Nanto Seed Co., Ltd., Nara, Japan. F_1_ seeds were planted and later self-pollinated. The resulting F_2_ was divided into two groups, one for a comparative test of two inoculation methods, pipetting and direct seed sowing methods, and the other for GRAS-Di map construction and QTL analysis. The 132 F_2_ plants were subjected to GRAS-Di analysis (see “*Linkage map construction using GRAS-Di technology*” for details) and further self-pollinated to produce the F_2:3_ (F_3_) populations. Among them, 126 and 130 F_2:3_ populations were used for QTL analysis conducted in 2021 and 2022, respectively. Ten seedlings of F_3_ per F_2:3_ population were used at each of the two periods of the inoculation tests that were done in the greenhouse. Okute-Sakurajima (JP 27228), the cultivar from which a radish reference genome sequence that was mainly used in this study, RSAskr_r1.0, was established (Shirasawa *et al.* 2020), was provided from the National Agriculture and Food Research Organization/NARO genebank, Japan to check its susceptibility to FOR.

### Inoculation test

All inoculation tests were performed using FOR MAFF731043 that was provided by NARO genebank, Japan. The potato sucrose broth (PSB) medium for fungus maintenance and inocula preparation was prepared using the protocol of Pu *et al.* (2012). Two methods of inoculation tests were employed in this study, the pipetting and the direct seed sowing methods (Supplemental Fig. 1). For the pipetting method, the experiments were conducted twice by Nanto Seed Co., Ltd., in Nara prefecture, Japan, in mid-June of 2021 and 2022. The inocula (2 × 10^5^ cfu) was added to each gram of soil, after transplanting ten-day old seedlings into plastic pots, and the plants were grown in the greenhouse where the minimum temperatures were 19.1°C and 16.0°C; the maximum temperatures were 32.9°C and 33.0°C; the average temperatures were 25.0°C and 23.5°C during the tests in 2021 and 2022, respectively. For the direct seed sowing method, the method followed the protocol of Pu *et al.* (2012), in which inocula was made by mixing healthy soil with diseased soil, in the ratio of 9:1, respectively. Thereafter, seeds were directly sown in the diseased soil, and the plants were grown in the growth chamber with temperatures controlled to a maximum range of 28°C with 16 h of light and 8 h of dark conditions. The phenotyping for QTL analysis was conducted using 10 F_3_ plants for each F_2:3_ population. Since this investigation required a large-scale experiment, the pipetting method was taken because it is less labor intensive than the direct seeding method. Inoculation trials of the parents and another set of the F_2_ plants were conducted using both methods to determine whether the results of the two methods were comparable to each other. In all the inoculation tests, RK15-1 was used as resistant control while AKM was used as susceptible control.

**Fig. 1.**
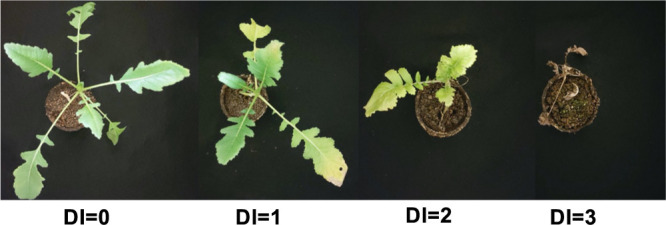
Inoculation test of fusarium yellows. Disease index score: the disease index (DI) ranged from 0 to 3; DI = 0 no disease symptom; DI = 1 showing slight yellowing of leaves; DI = 2 showing deep yellowing and loss of leaves/atrophy; and DI = 3 plant death. 0 ≤ DI ≤ 1.00 and 1.00 < DI ≤ 3.00 were classified as fusarium yellows resistance and susceptibility, respectively.

Disease severity index was taken at 28 days after inoculation (DAI) for all lines. The disease index (DI) ranged from 0 to 3, DI = 0, no disease symptom; DI = 1, showing slight yellowing of leaves; DI = 2, showing deep yellowing and loss of leaves/atrophy; and DI = 3, plant death (Fig. 1). Plants showing DI of 0 and average DI of 1 and below were classified as resistant, while others showing above 1 were classified as susceptible. When DI was calculated in phenotyping of F_2:3_ lines regarding QTL analysis, percentage of DI (PoDI) was estimated as follows, DI = Σ (DI × X) × 100/N × 3, where X = number of plants with symptom in each line, N = total number of seedlings. For the other inoculation test, the mean DIs (DI) were calculated in each experimental plot.

### Linkage map construction using GRAS-Di technology

For the construction of the linkage map using GRAS-Di, genomic DNA samples were extracted from the parents and the 132 F_2_ plants using NucleoSpin^®^ Plant II Kit (Takara Bio, Japan). After that, the first PCR was performed using arbitrary random primers containing Nextera adapter sequences and 3-base random oligomers that randomly bind to genomic DNA. Details of the PCR procedure can be found in Hosoya *et al.* (2019). The second PCR was conducted to add an Illumina multiplexed 8-base dual index to each amplicon pool. As a result, one sequencing library, including amplicons from 132 F_2_ plants and two replicates of each parent, were constructed and subsequently sequenced on the HiSeq 4000 platform (Illumina, USA) with 150-bp paired-end mode. Markers were identified with GRAS-Di software v 1.0.5 (Toyota, Aichi, Japan). The GRAS-Di procedure was conducted by Genebay Co., Ltd. (Kanagawa, Japan).

Sequencing the bulked GRAS-Di library using HiSeq 4000 technology generated 776 million reads, equivalent to 116 Gb of data, or 835 Mb per sample. Subsequent analysis using the GRAS-Di software resulted in a total of 3827 dominant markers, of which 31, 14, 1264, 2424, and 94 markers were classified into quality categories A, B, C, D, and E, respectively. Of these markers, those not classified as E (3733 markers) were further filtered based on the large skewness from the 3:1 ratio. The GRAS-Di software algorithm provides co-dominant polymorphisms between parents, as well as dominant polymorphisms specific to each parent. Since either type can be used for linkage-map construction, we constructed three genetic maps using these polymorphisms as markers: a map using only the segregation information of co-dominant markers, and two maps using both co-dominant markers and dominant markers from either the male or the female parent.

To generate F_2_ linkage maps, we used the Antmap ver.1.2 (Iwata and Ninomiya 2006) and excluded co-dominant and dominant markers with skewed segregation ratios at X^2^ test (P < 0.01 and P ≤ 0.05, respectively). For the construction of linkage groups, we employed the “all combinations” method and the “recombination” criterion, using a recombination threshold of 0.3 for the co-dominant and male-derived marker maps and 0.27 for the female-derived marker map. The parameters for locus ordering were “Kosambi map function”, “ordering SARF”, and “targeted groups all”.

### QTL detection and validation

QTL analysis was performed by composite interval mapping (CIM, Zeng 1994) using the Windows QTL cartographer v.2.5 (Wang *et al.* 2012) by combining the genotype (GRAS-Di) and phenotype (F_2:3_ inoculation test) data. CIM analysis was done using the Zmapqtl standard model 6, with a window size of 10 cM and a walking speed of 1 cM. To estimate a genome wide LOD threshold score for a QTL at 95% confidence level (P = 0.05), a 1000-permutation test was performed by shuffling the phenotypic means with the genotype (Doerge and Churchill 1996).

For validation of the two detected YR-QTLs on R2 and R7 chromosomes (designated as *ForRs2* and *ForRs1*), we selected four F_2_ plants (F_2_-13, F_2_-80, F_2_-50, and F_2_-95) with heterozygous genotypes at one QTL but homozygous at the other QTL. To determine the genotypes at the two QTLs, we referred to the genotypes of the flanking GRAS-Di marker information, AMP0000754 and AMP0003886 for *ForRs1* and *ForRs2*, respectively. Their F_3_ seeds ranging from 50 to 100 of each of the four genotypes were sown in the diseased soil for phenotyping (direct seed sowing inoculation method). At the same time, using seedling cotyledons or leaves, their genotypes were analyzed using the CAPS markers, AMP0000754-CAPS and AMP0003886-CAPS (Supplemental Table 1). The phenotypic data was analyzed using the Tukey-Kramer test of Excel Toukei 2010 for Windows (Social Survey Research Information Co., Ltd., Tokyo, Japan).

### Whole genome re-sequencing of the parents and genotyping

The parents were re-sequenced as follows. Their genomic DNAs were extracted using the NucleoSpin Plant II Kit (Takara Bio, Japan), then the DNA was sequenced by Genebay Co., Ltd. (Kanagawa, Japan) using the HiSeq 4000 device. The obtained reads were mapped to the radish reference genome sequence RSAskr_r1.0 (Shirasawa *et al.* 2020) by BWA (Li and Durbin 2010), and the nucleotide polymorphisms between the parent lines extracted from BAM files were identified using GATK (McKenna *et al.* 2010). The nucleotide polymorphisms between the parents were also visually identified using IGV software (Broad Institute). Based on the detected polymorphisms, CAPS and InDel markers located on the *ForRs1* region were designed for fine mapping (*ForRs1*-specific DNA markers; designated as #15-37, Supplemental Table 1).

For genotyping, one cm diameter pieces of fully expanded leaves or cotyledons were collected and frozen with liquid nitrogen for DNA extraction. The CTAB (Cetyl Trimethyl Ammonium Bromide) protocol, according to Murray and Thompson (1980) with modifications according to Sato *et al.* (2019), was used for extracting genomic DNA. The PCR genotyping was conducted as follows using DNA markers designed from the detected DNA polymorphisms between parents. The PCR mixtures were composed of 0.5 μl of plant DNA, 2.5 μl of EmeraldAmp PCR Master Mix (Takara Bio Inc., Japan), 0.2 μl of a mixture of forward and reverse primers (10 pmol each), and 1.8 μl of distilled water. The mixture was incubated at 94°C for 5 minutes, followed by 40 cycles of 94°C for 30 seconds (sec), 55°C–60°C for 30 sec, and 72°C for 30 sec and one cycle of 72°C for 5 min. For CAPS markers, the PCR products were digested with the appropriate restriction enzymes (Supplemental Table 1) before electrophoresis. Gel electrophoresis was conducted using either 1–2% agarose gel or 13% acrylamide gel to visualize the DNA fragments under a UV transilluminator. Gelstar solution (Takara Bio Inc., Japan) at a 10,000-fold dilution was used to stain acrylamide gels.

### Fine mapping of *ForRs1* using F_2:3_ populations

From 132 F_2_ plants, 7 plants (F_2_-15, 28, 46, 52, 61, 69, and 68) having recombination breakpoints within the *ForRs1* region were selected based on genotypes of two GRAS-Di markers, AMP0000754 and AMP0010720, which flank the *ForRs1* on R7 chromosome. The selected F_2_ plants were self-pollinated to produce F_3_ seeds. Next, 15 to 81 F_3_ plants of each F_2:3_ population were inoculated with FOR for a period of 28 days. At approximately 10 days after germination, each F_3_ plant was genotyped using *ForRs1*-specific DNA markers and only those having homozygous recombinant chromosomes were used for the subsequent fine-mapping study. To compare our identified *ForRs1* region with the location of the candidate genes identified by Ma *et al.* (2021), the reference genome of *R. sativus* cv. WK10039, Rs2.0, was used (Cho *et al.* 2022).

### Nanopore and RNA-sequencing

Nanopore sequencing of the YR parent, RK15-1 was performed to obtain precise genome sequence information of the *ForRs1* region of the resistant accession, as follows. DNA of RK15-1 was isolated using NucleoBond HMW DNA isolation kit (Takara Bio Inc., Japan), then sequencing adapters were ligated using Ligation Sequencing kit LSK110 according to the 1D protocol from Oxford Nanopore. The resulting library was then sequenced on MinION R9.4 flow cells (Oxford Nanopore Technologies, U.K.). The generated nanopore long reads were assembled into contigs using NECAT (Chen *et al.* 2021) and then were polished with RK15-1 Illumina short reads using NextPolish (Hu *et al.* 2020).

RNA-seq was also performed for both parents. The seedlings of each parent grown on the diseased and healthy soils were sampled at two different time points (8 and 11 days after sowing), then immediately immersed into liquid nitrogen and stored at –80°C until use. From each of the 8 samples, total RNA was extracted using RNeasy Mini Kit and RNA-Free DNase Kit according to manufacturer’s protocol (QIAGEN, Netherlands). For RNA-seq, RNAs of three seedlings at the same time point were pooled for each genotype. The resulting 8 total RNA samples were sent to Genebay Co., Ltd. (Kanagawa, Japan). The extracted RNA was purified using Sera-mag Magnetic Oligo (dT) Beads (Thermo Fisher Scientific, USA). The poly(A) selected RNA samples were used to prepare cDNA libraries and eight cDNA libraries were sequenced using Illumina HiSeq 4000 platform generating 150-bp paired-end reads. All RNA-seq reads from RK15-1 were pooled and mapped to the obtained nanopore contigs of RK15-1 using HISAT2 (v2.0.4), and subsequently analyzed by StringTie (v 1.2.3) (Pertea *et al.* 2016) to annotate transcripts and estimate their expression levels from the mapping data. Next, we also determined expression levels of the transcripts in each of the 8 samples using HISAT2 and StringTie. BLASTP searches were conducted using the predicted amino acid sequences of the annotated transcripts as queries against 2 databases, the TAIR 10 Proteins database from The Arabidopsis Information Resource (TAIR) (https://www.arabidopsis.org/index.jsp) and the radish reference genome RSAskr_r1.0 (https://plantgarden.jp/ja/index).

NGS sequence data have been submitted to the DDBJ database (https://www.ddbj.nig.ac.jp) under the accession number DRA016541 and DRA016542 for DNA-seq and RNA-seq, respectively.

## Results

### Segregation of YR in the progeny between RK15-1 and AKM

The two inbred lines, RK15-1 and AKM, along with their F_1_ hybrid and subsequent F_2_ population, were examined for susceptibility to FOR (Supplemental Fig. 2). RK15-1 showed DIs of 0.50 ± 0.25 and 0.50 ± 0.22 using the pipetting and direct seed sowing methods, respectively, indicating resistance to FOR. AKM, on the other hand, showed DIs of 2.70 ± 0.25 and 3.00 ± 0.00 using the pipetting and direct seed sowing methods, respectively, indicating susceptibility to FOR. The F_1_ plants were not stably resistant (DI = 1.18 ± 0.20) using the direct seed sowing method, suggesting the partial dominance of YR in this cross combination. The F_2_ plants showed bimodal frequency distribution of disease index in both methods. Additionally, cv. Okute-Sakurajima, the cultivar from which the reference genome sequence RSAskr_r1.0 was established (Shirasawa *et al.* 2020), was found to be susceptible to FOR (Average DI = 3.0 using 18 plants). Okute-Sakurajima exhibited wilt symptoms and the immediate death occurring 7 to 8 DAI. Conversely, AKM seedlings were moderately susceptible, displaying yellowing 13–16 DAI, followed by wilt and death (data not shown).

### Linkage map construction

GRAS-Di was conducted using 132 F_2_ plants between the parents. The obtained GRAS-Di markers, of which quality ranked from A to D, were further filtered to eliminate those exhibiting skewness from the Mendelian segregation within the F_2_. As a result, 3030 markers were classified into three categories: 363 co-dominant markers, 1345 dominant markers derived from RK15-1, and 1322 dominant markers derived from AKM. Using co-dominant markers, a map spanning 843.9 cM, with an average marker density of 2.32 cM was obtained (Table 1, Supplemental Fig. 3). The longest LG was 132 cM corresponding to R5, and the shortest LG was 63.3 cM corresponding to R3 and R7. The highest number of markers (59) was anchored on R2, followed by R5 (58), and the lowest number of markers (24) was identified on R7. The consensus linkage groups of the map were confirmed by aligning the GRAS-Di amplicon (Supplemental Table 2) to the reference genome of *R. sativus* (RSAskr_r1.0). Hereafter, the map will be referred to as “co-dominant derived map” in this article.

Although the co-dominant derived map has sufficient marker density for QTL analysis, to construct a map with higher density of markers, we attempted to use the information of dominant markers together with the co-dominant markers. Since combining RK15-1 (female)- and AKM (male)-derived markers into one map resulted in distorted marker order, such as large gaps and incorrect assignment of markers, possibly due to limitations of the mapping algorithm (Jansen 2009, Knapp *et al.* 1995, Tan and Fu 2007, Tan *et al.* 2017), the segregation data of RK15-1- and AKM-derived markers were separately combined with that of co-dominant markers. After the reduction of the numbers of the dominant markers that were mapped at the same position, the resulting RK15-1- and AKM-derived maps spanned 988.6 and 1127.5 cM, with average marker densities of 1.40 and 1.53 cM, respectively (Table 1, Supplemental Fig. 4). Hereafter, the 2 maps will be referred to as “RK15-1-derived map” and “AKM-derived map” in this article, respectively. The AKM-derived map was longer than the other two maps due to the increase of the length of the specific linkage groups such as the R3 and R7. The comparison between the three maps using co-dominant markers as anchor markers revealed good collinearity among them, although the order of some markers was reversed between the genetic maps (Supplemental Fig. 5). It is remarkable that AKM- and RK15-derived maps effectively positioned multiple dominant markers between two co-dominant markers, specifically, in the *ForRs1* region on the R7 chromosome, flanked by the co-dominant markers AMP0000754 and AMP0010720. This is particularly valuable for selecting recombinants with distinct chromosomal recombination points within this region for map-based cloning purposes.

### QTL analysis and confirmation of QTL effect

**Fig. 2.**
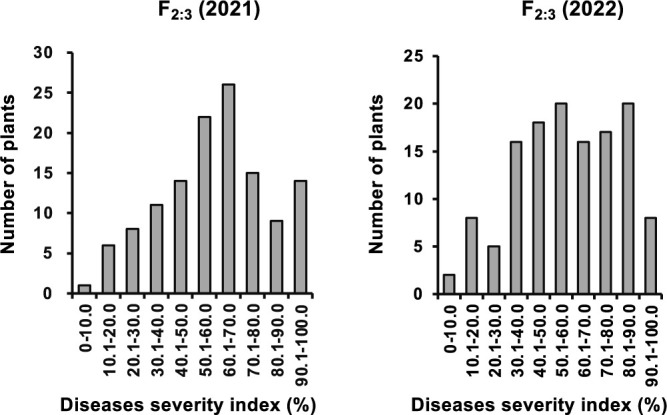
Frequency distribution of the percentage of disease severity index (PoDI) in the F_2:3_ populations. The results of the inoculation tests conducted in 2021 (left) and 2022 (right) were shown, respectively. The pipetting method was employed in these analyses.

Next, we generated F_2:3_ populations derived from the 132 F_2_ plants that were used in the GRAS-Di analysis. Among them, 126 and 130 F_2:3_ populations were used for inoculation tests conducted in mid-June 2021 and 2022 for QTL analysis. This population showed a continuous distribution of PoDI with a slight susceptibility shift between the two inoculation tests (Fig. 2). The distribution was skewed to higher PoDI in 2022 than in 2021, suggesting that symptoms were more severe in 2022 than in 2021.

**Fig. 3.**
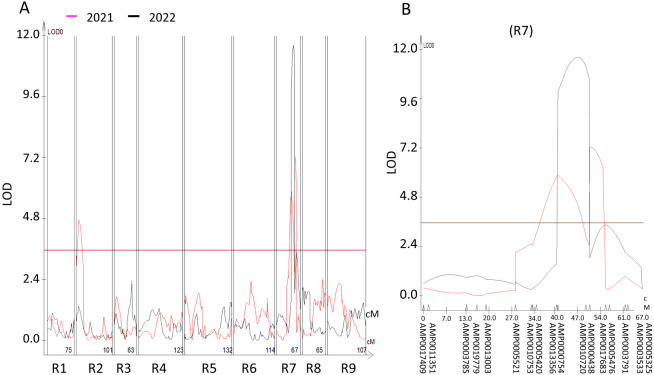
QTL profiles for YR derived in the F_2:3_ populations. Pink and gray lines in each graph represent data of 2021 and 2022, respectively. (A) LOD plots for YR along the whole chromosomes are shown. (B) LOD plots of R7 that harbors *ForRs1* are indicated. On the bottom of each graph, names of chromosomes (left) and GRAS-Di markers (right) are indicated. The horizontal lines in those graphs represent the threshold LOD values (3.6).

QTL analyses using the phenotype data of 2021 and co-dominant marker map detected two QTLs at R2 and R7 chromosomes, with LOD values of 4.7 and 7.3, respectively (Fig. 3). The QTL on R7 chromosome was repetitively detected when the phenotype data of 2022 was used, with LOD value of 11.6 (Fig. 3). This QTL on R7 accounted for 18.1 and 33.0 % of the phenotypic variation in 2021 and 2022, respectively (Table 2). Negative values for the additive effect of the two QTLs indicate that the RK15-1 allele in each of the R2 and R7 QTLs confers resistance to FOR. The major (R7) and minor (R2) QTLs were named *ForRs1* and *ForRs2*, respectively. Additional minor QTL on R5, together with the two QTLs, was detected in the 2021 trial when the RK15-1- and AKM-derived maps were used (Supplemental Fig. 6).

**Fig. 4.**
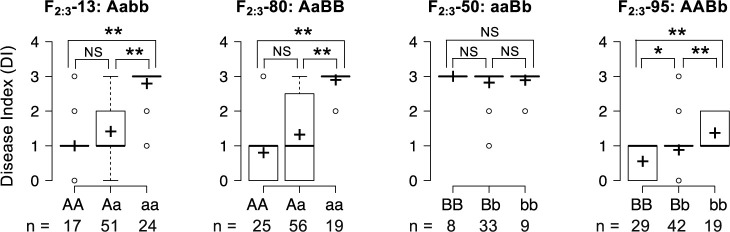
Relationship between YR phenotypes and genotypes at *ForRs1* and *ForRs2*. The values of DI of the F_3_ of the 4 investigated F_2:3_ populations, in which genotype segregation at either *ForRs1* or *ForRs2* locus occurs, are represented as box plots. At the top of each graph, the name of the F_2:3_ population and the F_2_ genotype are indicated. The genotypes at the segregating locus and the numbers of the investigated plants are indicated at the bottom of each graph. *, ** and NS indicate statistically significant at P ≤ 0.05, 0.01 and not significant, respectively.

To confirm the associations between 2 QTLs and YR, we chose four F_2_ plants (F_2_-13, F_2_-80, F_2_-95, and F_2_-50) with heterozygous genotypes at either one of the two QTLs, while homozygous at the other QTL. Two DNA markers, AMP0000754-CAPS and AMP0003886-CAPS, designed from GRAS-Di markers and RSAskr_r1.0 genome sequence that were found to be closely linked to each *ForRs1* and *ForRs2*, respectively, were used to predict the genotypes at the two QTLs (Supplemental Table 1). As a shorthand notation, parental allele genotypes at the *ForRs1* were assigned *A* and *a* for the resistance (RK15-1) and susceptibility (AKM) alleles, respectively. Similarly, parental alleles of *ForRs2* locus were assigned *B* (RK15-1) and *b* (AKM). Based on the analysis, the genotypes of F_2_-13, F_2_-80, F_2_-50, and F_2_-95 were identified as *Aabb*, *AaBB*, *aaBb* and *AABb*, respectively. These four F_2_ plants were then self-pollinated to produce segregating F_3_ for subsequent analysis. Different patterns of segregation in YR phenotypes were observed in the 4 F_3_ populations (Fig. 4). For F_2:3_-13 (*Aabb*) and F_2:3_-80 (*AaBB*) progenies segregating at the *ForRs1* locus, most of the plants with homozygous RK15-1 allele (*AA*) were resistant, but the plants with AKM homozygous allele (*aa*) were susceptible, regardless of the genotypes of *ForRs2* (Fig. 4). The susceptibility of the plants with heterozygous allele (*Aa*) in F_2:3_-13 and F_2:3_-80 fluctuated, and the respective average disease indexes (DIs) were 1.41 and 1.32 (Fig. 4). This result indicated that the RK15-1 allele of *ForRs1* (*A*) conferred higher resistance in a dominant or partial dominant manner, while the AKM allele (*a*) showed almost complete susceptibility. No contribution of the AKM allele of *ForRs1* (*a*) to YR was also supported by the observation of the F_2:3_-50 line (*aaBb*), in which all progenies showed susceptibility regardless of the genotypes at the *ForRs2* locus (*B*/*b*). Contribution of RK15-1-derived allele (*B*) at *ForRs2* to YR was observed in the F_2:3_-95 line (*AABb*), in which all plants were AA at *ForRs1*. These suggested epistatic interaction between the 2 loci.

### Fine mapping and comparison of the previously identified YR-QTLs

**Fig. 5.**
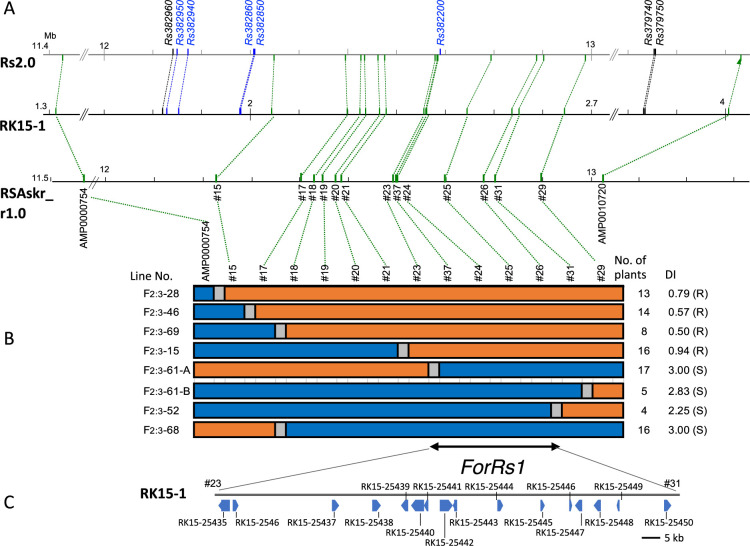
Fine mapping of *ForRs1* locus. (A) *ForRs1* overlaps *FoRsR7.1*. The schematic figure shows comparison between 3 alleles of the *ForRs1* locus of RK15-1 (contig-bctg00000035, this study), Okute-Sakurajima RSAskr_r1.0 (Shirasawa *et al.* 2020), and WK10039 (Rs2.0, Cho *et al.* 2022). Each of the 3 lines represents each allele. Positions and names of the DNA markers used in this study are indicated on RSAskr_r1.0, and the corresponding positions on the other 2 alleles were indicated with green dotted lines extending from them. Positions and the names of the genes associated with *FoRsR7.1*, that were identified by Ma *et al.* (2021), are indicated on Rs2.0. When the allelic sequences of the *FoRsR7.1* associated genes exist on RK15-1, their positions are indicated with dotted lines of blue and black extending from them as indicated on RS2.0. The genes highlighted in black and blue indicate the marker genes flanking *FoRsR7.1* and the candidate genes predicted by Ma *et al.* (2021), respectively. Please note that RK15-1 is resistant and Okute-Sakurajima is susceptible to FOR (this study), while YR phenotype of WK10039 is unknown. (B) Fine-mapped *ForRs1* region. Graphical genotypes and disease index (DI) of the selected recombinants in the F_2:3_ progenies are shown. Marker names are indicated at the top of the row. Homozygotes of the RK15-1-derived resistant allele and homozygotes of the AKM-derived susceptible allele are represented by orange and blue boxes, respectively. Regions of unknown genotypes due to marker intervals are shown as gray boxes. Name of lines and DIs are shown on the left and right side, respectively. The fine-mapped *ForRs1* region is indicated with a bi-directional line with arrow at the bottom. (C) Genes annotated in the fine-mapped *ForRs1* region of RK15-1. The genes in the region between #23 and #31 markers, with an estimated length of 195 kb, were represented as boxes.

F_2_ plants with chromosomal recombination within the *ForRs1* region (recombinants) were identified among the 132 F_2_ plants using the *ForRs1* flanking GRAS-Di markers, AMP0000754 and AMP0010720. As a result, 18 F_2_ plants having recombinants at the locus were found. Among them, 7 F_2_ recombinants (F_2_-15, 28, 46, 52, 61, 69, and 68) with distinct recombination points were used for the fine-mapping. The selected F_2_ recombinants had a non-recombinant (parental) and recombinant chromosome pair at *ForRs1* and were self-pollinated to produce the F_3_ progenies (Supplemental Fig. 7). Exceptionally, the F_2_-61 plant contained two recombinant chromosomes derived from both male and female gametes of the F_1_. Some of the F_3_ populations showed low variance in DIs, while others showed segregation (Supplemental Fig. 7). Simultaneously, genotypes in the *ForRs1* region of the F_3_ plants were determined using *ForRs1*-specific DNA markers designed from the re-sequenced data of the parents (#15-37, Fig. 5A, Supplemental Table 1). Based on the genotype data, we selected F_3_ plants having homozygous genotypes at all investigated markers for fine mapping, because they showed greater phenotypic stability of YR compared to those having heterozygous genotypes (Supplemental Fig. 7). The instability could be explained by the partial dominance of the resistant (RK15-1) to susceptible (AKM) allele that was inferred from the unstable resistant phenotype observed in the F_1_ (Supplemental Fig. 2). The resulting graphical genotypes of each F_2:3_ line, together with their DI, suggested that the *ForRs1* region could be narrowed down to the region between #23 and #31 markers (Fig. 5B).

### YR candidate genes in *ForRs1*

As described earlier, Okute-Sakurajima, the cultivar used to establish the radish reference sequence RSAskr_r1.0 (Shirasawa *et al.* 2020) which was mainly used in this study, was susceptible to FW. Since it was not clear whether the reference genome of the susceptible accession was useful for identification of YR candidate genes in the *ForRs1* region of RK15-1, we conducted long-read whole genome sequencing of RK15-1 using Nanopore technology to obtain the genome sequence information of the resistant accession. From the analysis, we obtained 666 contigs with the maximum and minimum contig sizes of 12.9 Mbp and 0.53 Mbp, respectively (Supplemental Table 3). The total assembly size of 489 Mbp was roughly equal to the estimated radish genome size of 504.5 Mb (Shirasawa *et al.* 2020). The completeness of the assembly evaluated using BUSCO (Manni *et al.* 2021) was 99.2%, high enough to be used for the downstream analyses. From the contigs, contig-bctg00000035 (4.5 Mb) was identified to contain both sequences of AMP0000754 and AMP0010720 that flank *ForRs1*. The contig showed high level of sequence collinearity with the allelic region of RSAskr_r1.0 (Fig. 5A), again suggesting accuracy of the sequence assembly. Based on the contig-bctg00000035, the length of the fine-mapped *ForRs1* region (between #23 and #31 markers) was estimated as 195 kb (Fig. 5B, 5C).

Previously, Lee *et al.* (2021) conducted QTL analysis using an F_2_ population derived from a YR line and a susceptible line and reported that the *qFWR3* detected on R7 was co-located with the SNP located in Rs404770 gene, which encodes the glycosyltransferase family protein. The blastn search using Rs404770 sequence as a query revealed that this gene is not located in the *ForRs1* region of this study that is sandwiched by AMP0000754 and AMP0010720. Ma *et al.* (2021) detected a YR-QTL, *FoRsR7.1* on the chromosome R7, which was defined by a gene Rs382960 and a DNA marker BRPGM1176 on a radish reference genome sequence Rs1.0. They identified 5 R-genes in this region, including 3 candidate genes for YR. Therefore, we tried to know relative positions of *ForRs1* and *FoRsR7.1*. Since the sequence of BRPGM1176 marker was not available, we used Rs379750, a gene close to BRPGM1176, to determine the *FoRsR7.1* region. Fig. 5A showed high level of collinearity between the sequences of Rs2.0 (newer version of Rs1.0, Cho *et al.* 2022), RSAskr_r1.0 (Okute-Sakurajima), and contig-bctg00000035 (RK15-1) at *ForRs1* region. This comparison revealed that the fine-mapped *ForRs1* region was completely included in the *FoRsR7.1* region (between Rs382960 and Rs379750). However, 2 out of the 3 candidate genes in *FoRsR7.1*, Rs382940 and Rs382950 were outside of the fine-mapped *ForRs1* region. Furthermore, the other candidate gene in *FoRsR7.1*, Rs382200, was not present in the contig-bctg00000035 of RK15-1. These findings suggest that YR genes in the *ForRs1* region of this study could be different from any of the 3 candidate genes in *FoRsR7.1* (Ma *et al.* 2021), even though the two regions are completely overlapped.

In the fine-mapped *ForRs1* region on contig-bctg00000035 of RK15-1, 16 genes were annotated based on the RNA-seq data and included catalytic enzymes, receptor-like proteins, a transmembrane protein of unknown function, and various proteins belonging to different families or with specific roles such as transcription factors and ribosomal proteins (Fig. 5C, Supplemental Table 4). We excluded 8 genes (RK15.25438, RK15.25439, RK15.25443, RK15.25445, RK15.25446, RK15.25447, RK15.25449, RK15.25450) from possible candidate genes of *ForRs1* because they showed no sequence variation of the coding regions between the parents, and they were stably expressed in both RK15-1 and AKM (Supplemental Table 5, Supplemental Fig. 8).

Among the remaining eight candidate genes (Table 3), in AKM, the RK15.25441 mRNA, annotated as a RING/U-box superfamily protein (E3 ligases), exhibits an abnormally truncated structure (Supplemental Fig. 9) compared to that of RK15-1. This suggests that while the gene mutation in the susceptible AKM parent may be linked to YR-susceptibility, the CDS sequence of the susceptible Okute-Sakurajima gene (RSAskr1.0R7g74443) with the same annotation perfectly matches RK15-1 CDS. This indicates that the RK15.25436 gene may be excluded from the list of candidates. Compared to RK15-1, two AKM esterase genes (RK15.25436, RK15.25437) and one AKM casein lytic proteinase gene (RK15.25440) expressed truncated mRNAs. These genes lack R-gene specific motifs, suggesting that their role as hydrolytic enzymes makes it unlikely for them to function as resistance genes.

The remaining 4 genes encode proteins that were assumed to be related to pathogen resistance. RK15.25435 (RLP) possesses R-protein-like motifs such as leucine-rich repeats (LRRs), where many SNPs were found between RK15-1 and the two susceptible lines, AKM and Okute-Sakurajima. Consequently, premature stop codons were generated in the coding sequences (CDSs) of the two susceptible lines (Supplemental Fig. 10, Table 3). RK15.25448 encodes a receptor-like kinase (RLK) which possesses a leucine-rich repeats (LRRs) and a kinase domain. A nonsynonymous substitution (E>Q) was found in the exon 1 of this gene between RK15 and the two susceptible lines. Additionally, another nonsynonymous substitution (L>P) was found in exon 2 of AKM allele. RK15.25442 encodes a tetratricopeptide repeat (TPR)-like superfamily protein, and RK15.25444 encodes a putative transcription factor MYB95 which contains a Myb DNA-binding domain. Those protein families are known to regulate various physiological processes including pathogen resistance. In these two genes, nonsynonymous substitutions were found between RK15-1 and the two susceptible lines (Supplemental Fig. 10). As a result, these four genes remained as the best candidates for *ForRs1*.

## Discussion

### Linkage map construction

The GRAS-Di software algorithm provides co-dominant polymorphisms between parents, of which the number is limited, whereas it provides thousands of dominant markers (Enoki 2019, Enoki and Takeuchi 2018, Miki *et al.* 2020). Thus, use of dominant markers to map construction is important for GRAS-Di technology. However, it is reported that estimating recombination frequencies between dominant markers is least efficient in F_2_ progeny, particularly in repulsion dominant markers (Jansen 2009, Knapp *et al.* 1995, Tan and Fu 2007, Tan *et al.* 2017). To overcome this challenge, Miki *et al.* (2020) and Fekih *et al.* (2023) mapped the GRAS-Di amplicon sequences to the reference genome sequence to revise irregular marker orders, and thereafter, the recombination frequencies of the dominant markers were estimated by R/qtl software. As a result, such a reference genome mapping-based genotyping method successfully generated high-quality reference genomes. Alternatively, to address the challenge posed by the unsuitability of repulsion dominant markers for linkage maps, Knapp *et al.* (1995) suggested that dominant markers can be split into two groups and mapped, one with dominant alleles from the male and one with dominant alleles from the female, thereby creating two pure-coupling F_2_ maps. Therefore, we created separate maps, and consequently, successfully created a set of high-density GRAS-Di maps by separately including RK15-1 (female)-derived and AKM (male)-derived markers in co-dominant markers. Those maps effectively positioned multiple dominant markers between two co-dominant markers in the *ForRs1* region on the R7 chromosome, which was very useful for selecting recombinants with distinct recombination points in the target region. It is thought that the use of GRAS-Di would be promoted by effectively using dominant markers. In fact, in the F_1_ pseudo-testcross method applied to heterozygous perennials, where the use of uniparental dominant markers is preferred over co-dominant markers, high-density male and female linkage maps using GRAS-Di markers have been facilitated in sugarcane (Umeda *et al.* 2021) and apple (Moriya *et al.* 2021).

### Comparison of the previously identified YR-QTLs in radish

Several studies have discovered YR-QTLs in different genomic regions of radish. However, the absence of shared markers among researchers makes it difficult to confirm the identical nature of the identified QTLs. To address this issue, we used the consensus linkage group (Shirasawa and Kitashiba 2017). Regarding the minor YR-QTLs, in the study conducted by Ma *et al.* (2021), two minor YR-QTLs, namely *FoRs9.1* and *FoRs9.2*, were identified on R9, appearing only once across three studies. Yu *et al.* (2013) reported the discovery of eight YR-QTLs (*qFW1*-*8*) during three trials spanning a two-year period. Among these, a major YR-QTL was identified on LG3 (R5), while the remaining seven QTLs, *qFW6* on LG4 (R4), *qFW3* on LG3 (R5), *qFW1* and *qFW2* on LG2 (R6), *qFW5* and *qFW8* on LG7 (R8), and *qFW7* on LG6 (R9), were classified as relatively minor YR-QTLs based on the R^2^ values. In another study by Lee *et al.* (2021), a total of four YR-QTLs were identified, with three QTLs on R3, R6, and R8 being categorized as minor YR-QTLs. Interestingly, the syntenic analysis revealed a correspondence between *FoRs9.1* and *qFW7* on LG6 (R9) (Ma *et al.* 2021). Thus, the reported minor YR-QTLs distributed in the chromosomes spanning R3 to R9 indicated that our study could discover a novel minor YR-QTL, *ForRs2*, located on R2.

Regarding the major YR-QTLs, Kaneko *et al.* (2007) found a YR-QTL on LG1 (R7), corresponding to the long arm of chromosome 1 in *A. thaliana*. Yu *et al.* (2013) discovered *qFW4* on LG3 (R5), which explained the majority of the phenotypic variation. Ma *et al.* (2021) identified two major YR-QTLs, *FoRsR7.1* and *FoRsR9.3*, on R7 and R9 chromosomes, respectively. Lee *et al.* (2021) performed a QTL analysis and associated one of the identified QTLs with an SNP on R7 from their genome-wide association study (GWAS). Thus, major YR-QTLs conferring high resistance to FOR have been detected in R5, R7, and R9, with R7 being repeatedly identified by different investigators. Comparison of the location of the major YR-QTLs is also difficult due to the lack of common markers among the investigators. Even so, in this study, we showed that the region of *ForRs1* overlapped with *FoRsR7.1* of Ma *et al.* (2021), suggesting the possibility that the region could be one of the major loci for YR in radish. However, since the candidate genes identified by the 2 groups differ from each other, together with the allelic structural diversity at the locus that was observed as the presence-absence variation of a gene (Rs382200) between alleles, it is not clear if the YR genes in *ForRs1* and *FoRsR7.1* are the same or different. Plant disease resistance loci often show diversity in allelic structure resulting from rapid genomic changes such as deletions, duplications, and gene losses/exchanges, which may have contributed to the rapid evolution of the disease resistance genes (Kuang *et al.* 2005, Read *et al.* 2020). Therefore, despite the availability of several radish reference genome sequences, if they were derived from susceptible accessions such as Okute-Sakurajima of RSAskr_r1.0, it is unknown whether their information is useful to molecularly determine the resistance gene. This is because the susceptible allele of the resistance gene cannot be annotated when it has accumulated nonsense mutations or when the gene sequence is completely lost in the susceptible allele. Regarding this, we believe that it is quite important to have a long contig sequence of the genomic region of interest for the resistant accessions/varieties which would be used in specific mapping study. In this study, we used Nanopore sequencing technology to obtain the long genome sequenced of the *ForRs1* region in the YR parent, RK15-1.

### Identification of the candidate genes controlling YR

In this study, *ForRs1* region fine-mapping and RNA-seq analysis was performed, resulting in the identification of 16 transcripts. By utilizing the RNA-seq data and nucleotide sequences of each potential gene, eight genes that exhibited no sequence variation between YR RK15-1 and susceptible AKM were excluded from consideration as candidate genes. Among the remaining eight candidate genes listed in Table 3, after excluding the genes expressing hydrolase and ligase enzymes, four R-gene analogue genes with significant non-synonymous SNPs between RK15-1 and AKM emerged as the most promising candidates.

RK15.25448 encodes an RLK domain similar to the *A. thaliana* resistance gene PBS1-Like 29 (AT1G74490). PBS1-Like proteins are known to associate with RPS5 (RESISTANCE TO PSEUDOMONAS SYRINGAE 5) and confer resistance against certain strains of *Pseudomonas* *syringae* in *A. thaliana* (Pottinger and Innes 2020). The PBS1-Like genes belong to the Arabidopsis receptor-like cytoplasmic kinase (RLCK) subfamily VII, which consists of 46 members. Among the PBS1-Like orthologous genes in the radish genome, it is necessary to investigate whether RK15.25448, the ortholog of PBS1-Like 29, exhibits specificity in recognizing *F*. *oxysporum* f. sp. *raphani*. Another candidate resistance gene, RK15.25435, encodes an RLP (receptor-like protein) with a leucine-rich repeat (LRR) motif. Interestingly, the YR radish candidate gene (*Fwr1*) identified on chromosome R5 (Yu *et al.* 2020) is also an *RLK*. Notably, albeit *B. oleracea* and *B. rapa* are closely related to radish, their TIR-NBS-LRR genes *FocBo1* and *FocBr1*, which are not *RLP*/*RLK* genes, confer Fusarium wilt resistance, respectively (Shimizu *et al.* 2014, 2015).

The gene RK15.25438, which encodes a protein interacting with phytosulfokine, was not considered a candidate gene due to the identical coding sequence (CDS) in RK15-1 and AKM, as well as their nearly identical mRNA expression levels. Despite its exclusion, RK15.25438 possesses an LRR domain similar to PSKR1, which is involved in plant immunity and interacts with a danger-associated molecular pattern, phytosulfokine (Hu *et al.* 2023). The alignment of the amino acid sequences of the LRR domains of PSKR1 and RFO2 (encoded by the fusarium resistance gene RFO2 in *A. thaliana*) showed a high similarity, and RFO2 acts as a decoy receptor for PSKR1 (Shen and Diener 2013). The alignment of amino acid sequences, including RK15.25438, revealed a high similarity among these three gene products (Supplemental Fig. 11). Thus, the RK15.25438 gene might have an undisclosed role in disease resistance reactions. As just mentioned, AKM has the functional allele of this gene, but Okute-Sakurajima (FW susceptibility) does not because of the deletion of the region containing this gene, which was confirmed by the sequence analysis of the *ForRs1* region of Okute-Sakurajima. We identified three RLP/RLK genes in the *ForRs1* locus, including this gene. Duplicated R-genes are reported to commonly co-localize with disease resistance QTL. Previous studies have shown that clusters of NBS-LRR genes can provide effective resistance against rice blast (Ashikawa *et al.* 2008) and *Peronospora parasitica* in Arabidopsis (Sinapidou *et al.* 2004). Furthermore, it has been found that the rice CC-NLR pair RGA4 and RGA5, derived from the tandemly repeated genes, form a heterodimer in response to the Avr-Pia effector of *Magnaporthe oryzae* (Cesari *et al.* 2014).

We also identified RK15.25442 (tetratricopeptide repeat (TPR)-like superfamily protein) and RK15.25444 (putative transcription factor MYB95) in the delimited region. The former protein has a protein-protein interaction domain by which the protein can interact with and regulate the activity of key immune proteins, such as resistance (R) proteins. In fact, MoChia1, a chitinase secreted by rice pathogen *M. oryzae*, suppresses plant immune response by binding chitin. Rice protein OsTPR1 interacts with MoChia1, allowing chitin accumulation and re-establishing immunity, demonstrating how plants counteract fungal chitinase (Yang *et al.* 2019). Regarding MYB transcription factor, in sorghum infected by the fungal pathogen *Colletotrichum sublineolum*, accumulation of 3-deoxyanthocyanidin phytoalexins at the site of primary infection is regulated by a MYB transcription factor (Ibraheem *et al.* 2015). The RK15.25442 and RK15.25444 genes exhibited polymorphism between RK15-1 and AKM. Therefore, it is necessary to further examine whether RK15.25442 and RK15.25444 genes qualify as candidate genes.

The present study pioneers the utilization of a GRAS-Di map for constructing the genetic map of cruciferous crops and achieving a detailed mapping of the YR-QTL on the R7 chromosome of Japanese radish. As a result, we successfully narrowed down the *ForRs1* locus to a 195 kb region and identified four genes including RLP/RLK genes as best candidate genes. These findings will provide groundbreaking insights into radish YR breeding and understanding the genetic mechanism of YR. Further studies are necessary to determine the responsible genes for YR at the *ForRs1* locus.

## Author Contribution Statement

C.S.A.E., J.S., E.F. and K.O. conceived and designed the research. C.S.A.E., S.K., M.K. and K.O. conducted the experiments and prepared samples. T.K., M.S., M.N. and N.M. assisted in NGS library preparation and sequencing. C.S.A.E., T.K., M.S, M.N. and K.O. analyzed the data. C.S.A.E., C.O.E., E.F. and K.O. wrote the manuscript. E.F. and K.O. supervised the experiments. K.O. contributed by providing funding and also managed the whole project. All authors contributed to the development of this manuscript.

## Supplementary Material

Supplemental Figures

Supplemental Table 1

Supplemental Table 2

Supplemental Table 3

Supplemental Table 4

Supplemental Table 5

## Figures and Tables

**Table 1.** Linkage group data for the F_2_ population resulting from the crossing of RK15-1 and AKM. Length in centimorgan and number of markers are indicated at the top and bottom of each row for each linkage group, respectively.

Maps	Linkage group									Total
	R1	R2	R3	R4	R5	R6	R7	R8	R9	
Co-dominant map	74.6	100.7	63.3	123.4	132.0	114.4	63.3	64.8	107.4	843.9
	(32)	(59)	(30)	(30)	(58)	(46)	(24)	(41)	(43)	(363)
RK15-1-derived map + co-dominant marker	95.5	120.9	90.2	138.1	145.3	130.4	80.8	77.7	109.7	988.6
	(52)	(96)	(71)	(87)	(100)	(100)	(57)	(79)	(63)	(705)
AKM-derived map + co-dominant marker	80.0	139.1	158.3	132.4	135.6	130.6	117.0	100.4	134.1	1127.5
	(57)	(99)	(100)	(81)	(84)	(94)	(61)	(85)	(77)	(738)

**Table 2.** Summary of QTLs detected for yellows resistance to *Fusarium oxysporum* f. sp. *raphani*

Name	Linkage Group		Marker interval	1 LOD confidence interval (cM)	LOD	R^2^ (%)*^a^*	Additive effect*^b^*	Dominance effect
*ForRs2*	R2	1st Test	AMP0010176–AMP0013639	2.54–15.88	4.7	11.5	–8.9	–6.2
*ForRs1*	R7	1st Test	AMP0000754–AMP0009342	41.1–54.8	7.3	18.1	–12.1	–2.7
		2nd Test	AMP0000754–AMP0010720	41.1–50.7	11.6	33.0	–17.5	–4.2

*^a^* Proportion of the phenotypic variance explained by each QTL. *^b^* Additive effects of RK15-1 allele.

**Table 3.** Annotation, parent polymorphism, and expression level in the candidate genes located in the delimited region of *ForRs1*

Genes	Annotation	Mutations found in AKM referred to the amino acid sequences of RK15-1				TPM*^a^*			
		Structure of transcript	Premature stop codon	No. of substituted amino acid residues	No. of deleted/inserted amino acid residues	RK15-1		AKM	
						CONT	INF	CONT	INF
RK15.25435	Receptor like protein 15	Normal	Yes (by frameshift at F507fs)	19 (prior to the pre-mature stop codon)*^b^*	2 (W85_A86insAR) 1 (E398_S399insI)	0.65	1.59	0.09	0.77
RK15.25436	Encodes a member of the glycerophosphodiester phosphodiesterase (GDPD) family	Abnormal*^c^*	No	14 (L7S T16N G33S F92A T93I N98D S102N N180S G193S K221R T240I A290T E315Q QS371T)	8 (N238_L239insLIDSPKVL)	19.37	25.46	9.34	15.43
RK15.25437	Encodes a member of the glycerophosphodiester phosphodiesterase (GDPD) family	Abnormal	No	8 (V20G S118F M120L Y149F P191S A238D V269M H386L)	0	4.39	3.15	4.03	4.79
RK15.25440	Encodes ClpB1, which belongs to the casein lytic proteinase/heat shock protein 100 (Clp/Hsp100) family	Normal	No	1 (Q59A)*^d^*	1 (E58_Q59insT)	4.66	4.86	5.54	9.79
RK15.25441	RING/U-box superfamily protein	Abnormal	No	4 (P28A S108N N134S Q142E)	12 (F13_K14insKSMGEDTVIEPI) 1 (V137del) 1 (D147del)	3.26	3.75	0.43	0.12
RK15.25442	Tetratricopeptide repeat (TPR)-like superfamily protein	Normal	No	1 (S10F)	0	3.83	4.00	4.16	2.71
RK15.25444	Encodes a putative transcription factor (MYB95)	Normal	No	1 (N131S)*^d^*	0	0.29	0.15	1.91	1.15
RK15.25448	Protein kinase superfamily protein. PBL29, PBS1-LIKE 29	Normal	No	2 (E30Q L116P)	0	0.77	0.75	0.13	0.00

*^a^* TPMs (Transcripts per million) were obtained in the RNA-seq analysis using RNAs collected at the 11 days after sowing from three seedlings of each parent grown on the diseased (INF) and healthy (CONT) soils. *^b^* Substituted amino acid residues are indicated in Supplemental Fig. 12. *^c^* Visible structural abnormality observed using IGV, as exemplified in Supplemental Fig. 9. *^d^* Similarity percentage of the proteins between RK15-1 and AKM is 100%.
