# Supplementary material for: Quantitative trait locus (QTL) analysis and fine-mapping for Fusarium oxysporum disease resistance in Raphanus sativus using GRAS-Di technology
Source: Breed Sci. 2023 Nov 1;73(5):421–34. doi: 10.1270/jsbbs.23032 (PMC11082455; doi:10.1270/jsbbs.23032)
Supplement: Supplementary file 1 — Supplemental Figures [file 73_421_s1.pdf]

Ezeah et al./Breeding Science
doi: 10.1270/jsbbs.23032

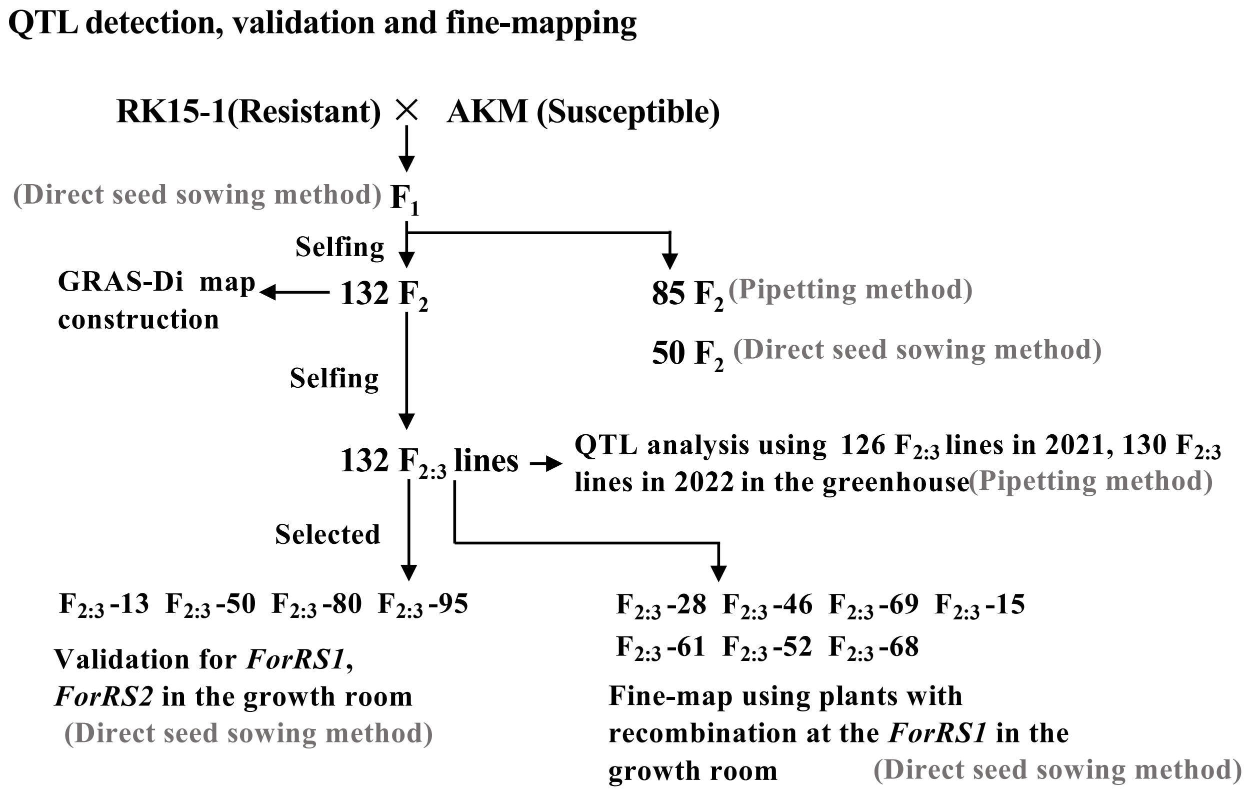

Supplemental Fig. 1. Schematic diagram showing the materials and inoculation methods used in this study. The development process of $F_{2:3}$ lines derived from the cross between RK15-1 and AKM inbred lines of *R. sativus.* Inoculation test was performed using pipetting or direct seed sowing method or both

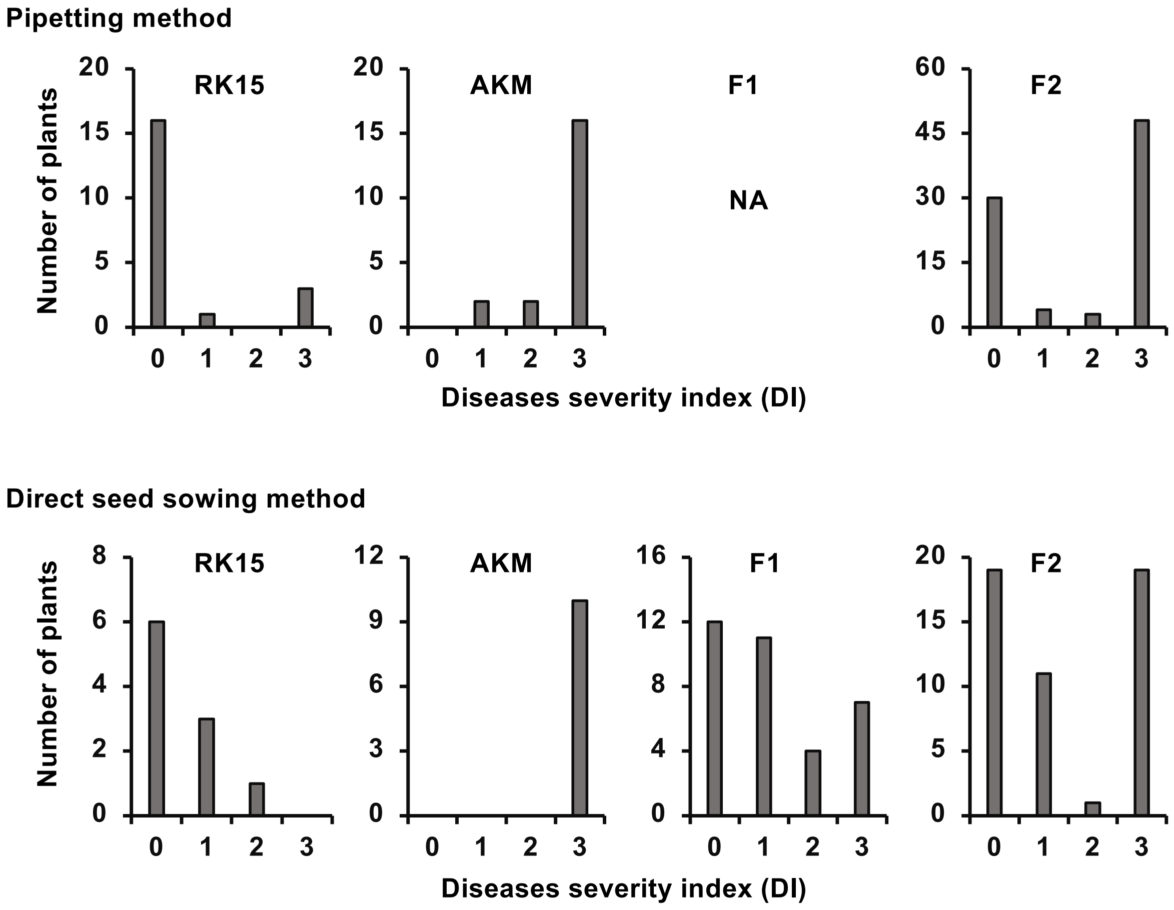

Supplemental Fig. 2. Frequency distribution of disease severity index (DI) for the parental inbred lines, RK15-1 and AKM, their F_1 and F_2 progenies. For the inoculation tests, pipetting and direct seed sowing method were used in the greenhouse and the growth room, respectively.

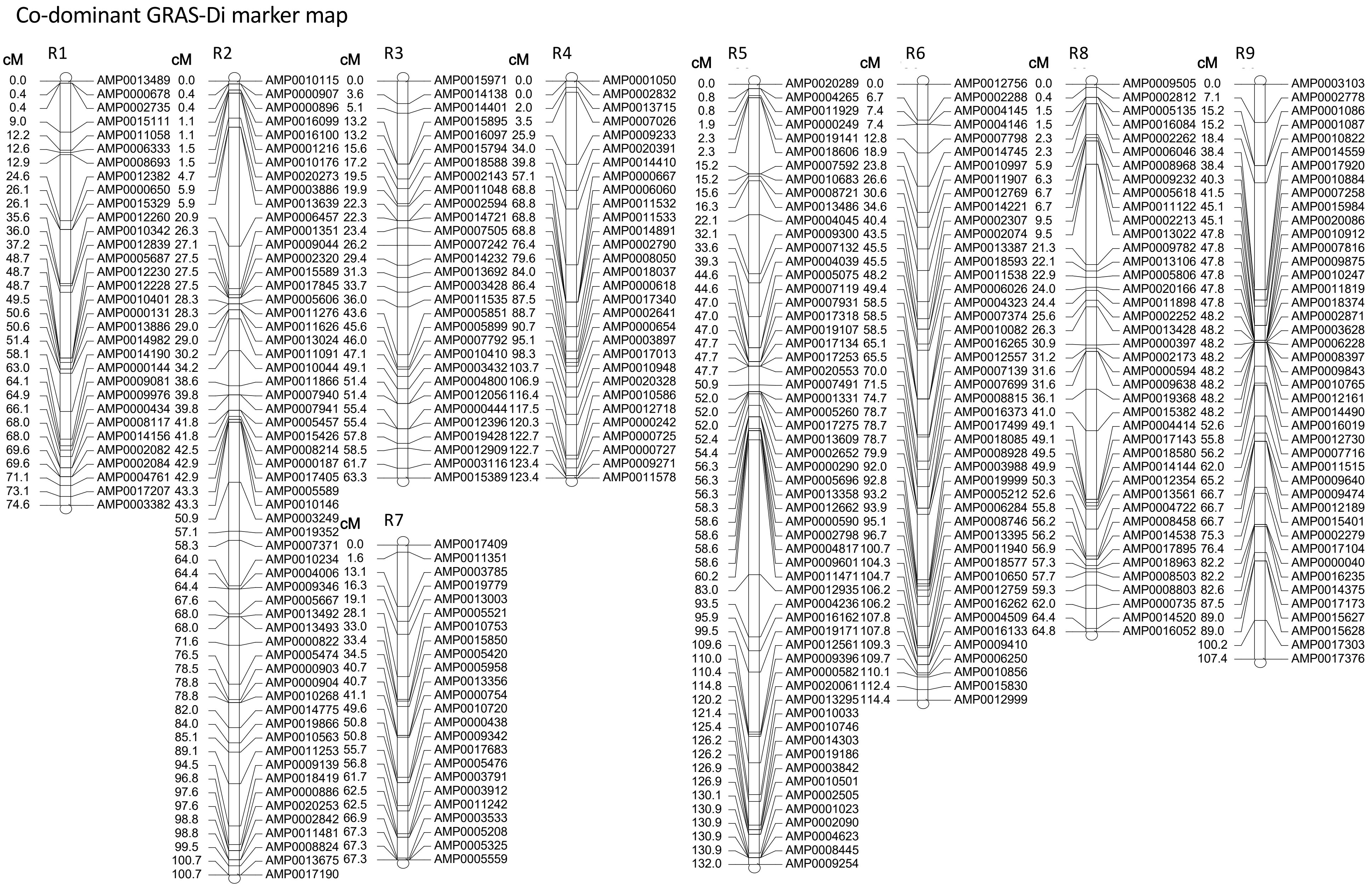

Supplemental Fig. 3. A linkage map constructed using co-dominant GRAS-Di markers. Marker position in centimorgan (cM) and locus of ordering are indicated on the left and right side of the linkage groups, respectively.

(A) RK15-1 (female)-derived map

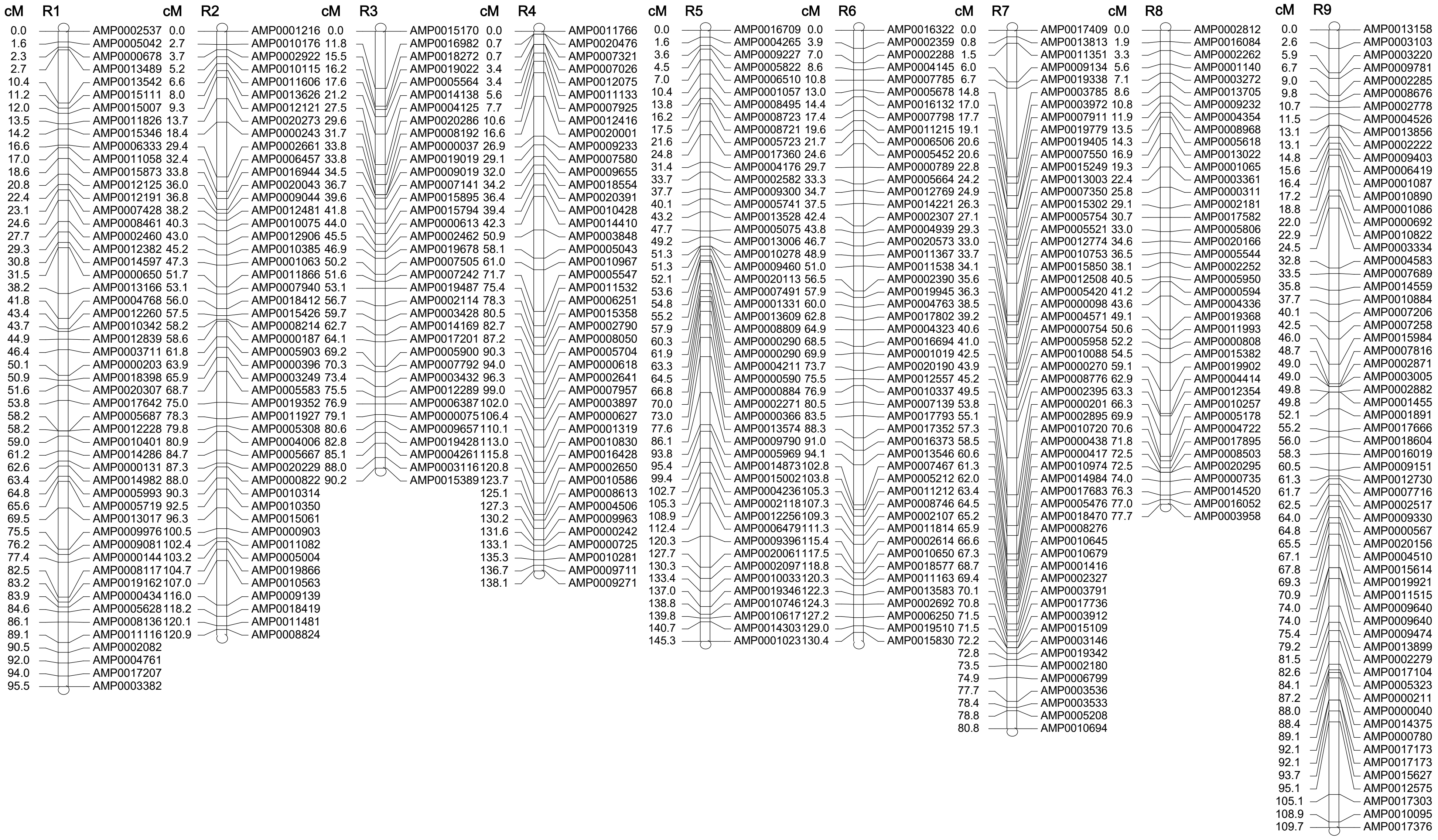

Supplemental Fig. 4. Linkage maps constructed using co-dominant and dominant markers. (A) RK15-1 (female)-derived map, (B) AKM (male)-derived map.

(B) AKM (male)-derived map

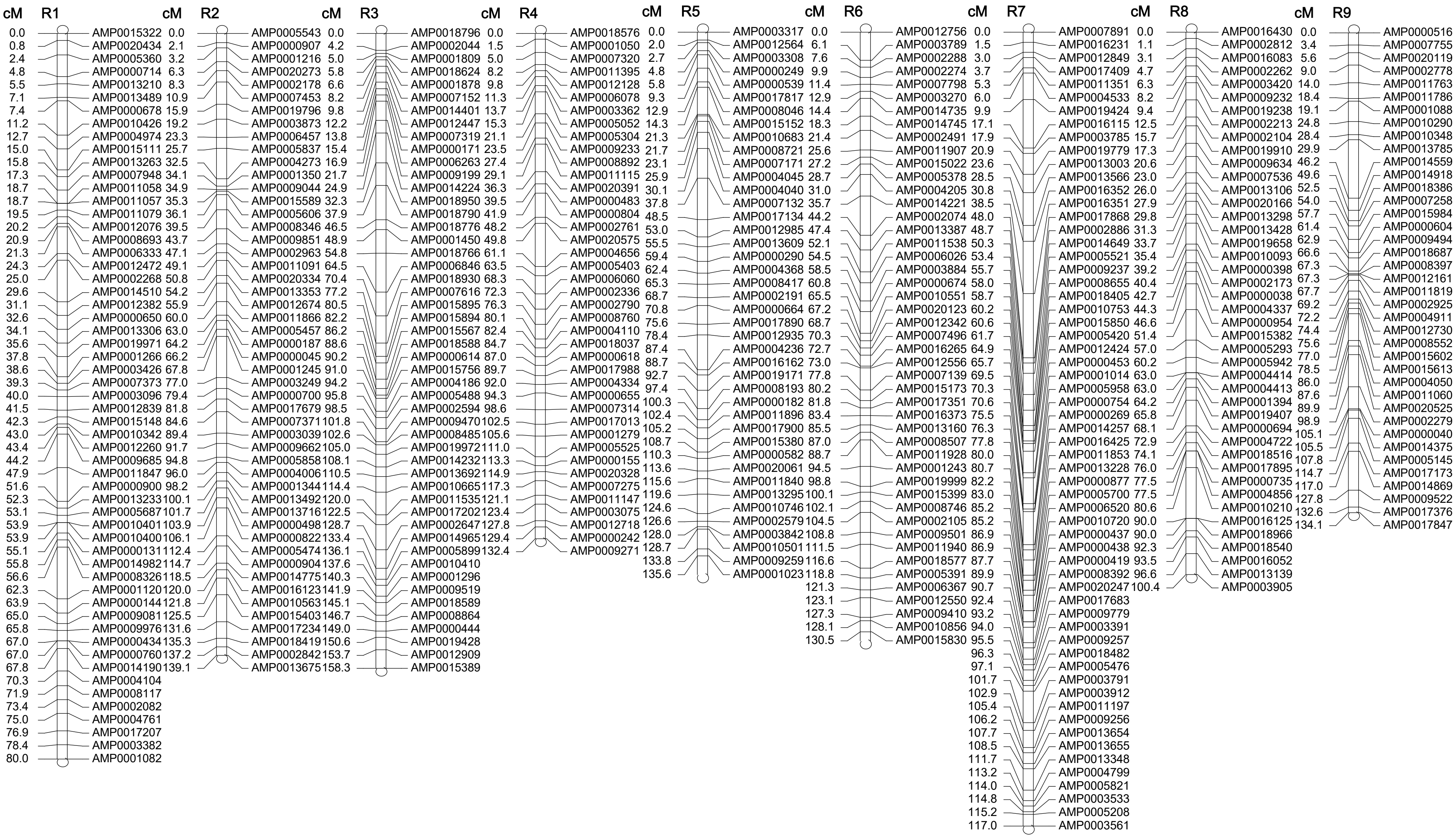

Supplemental Fig. 4. Linkage maps constructed using co-dominant and dominant markers. (A) RK15-1 (female)-derived map, (B) AKM (male)-derived map.

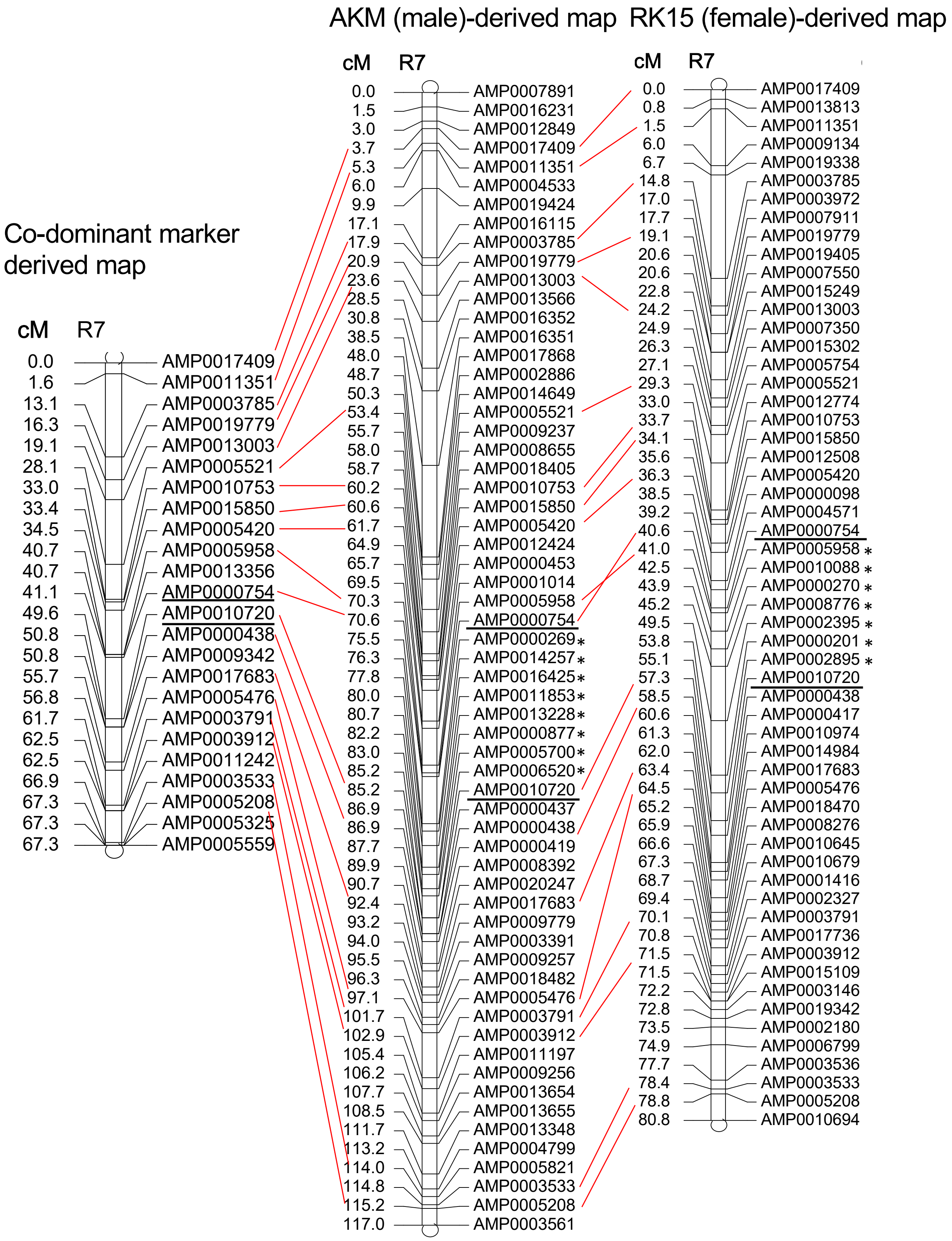

Supplemental Fig. 5. Alignment between R7 linkage groups of the 3 linkage maps. Red lines connecting between 3 maps indicate anchor co-dominant markers. Underlined markers are co-dominant markers that flank *ForRs1* region.

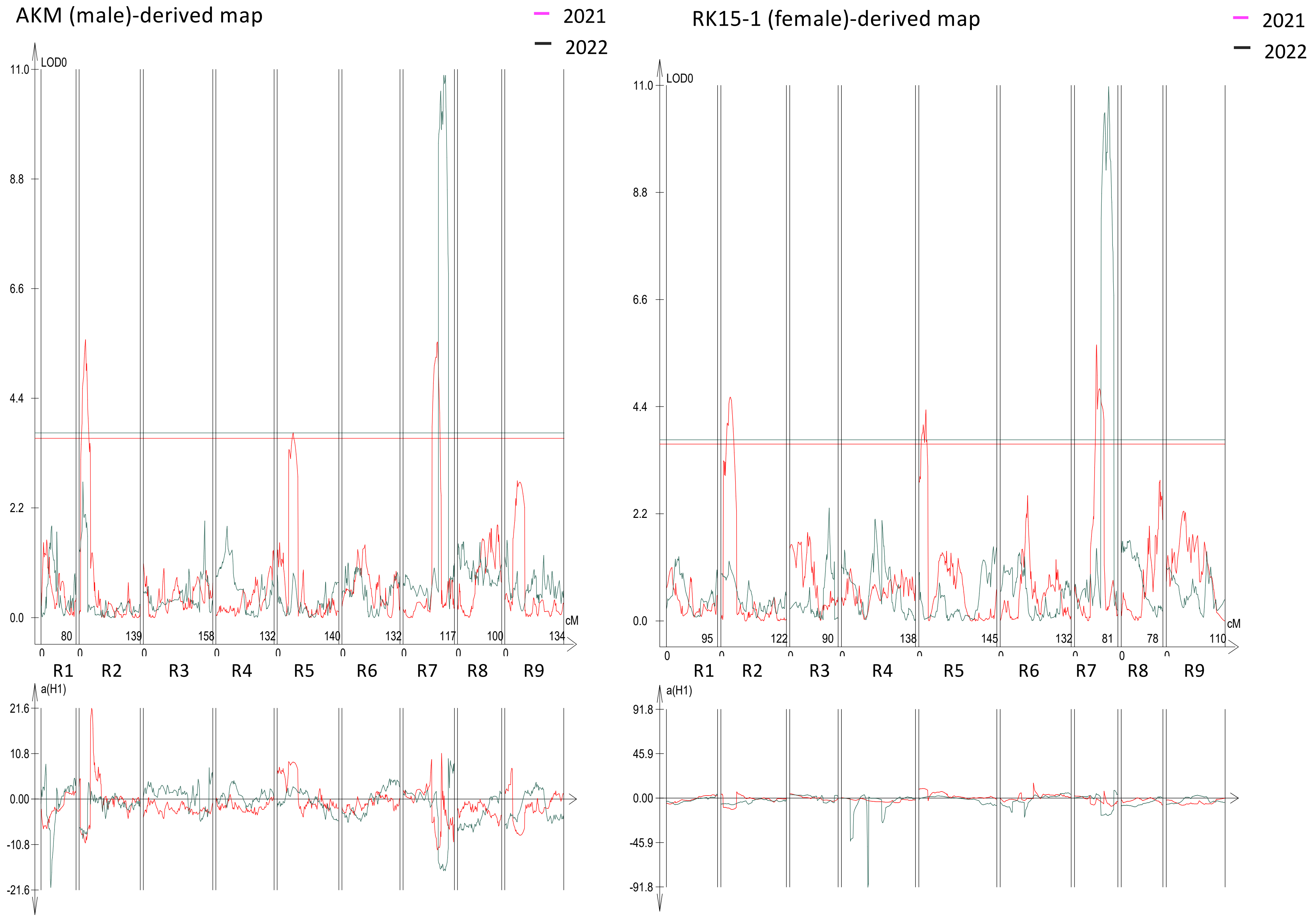

Supplemental Fig. 6. QTL profiles for YR using AKM- and RK15-1-derived map. Pink and gray lines in each graph represent data of 2021 and 2022, respectively. On the left, LOD plots for YR drawn using AKM-derived map is shown. On the right, LOD plots for YR drawn using RK15-1-derived map is shown. The horizontal lines in those graphs represent the threshold LOD values (3.5 in 2021 and 3.7 in 2022).

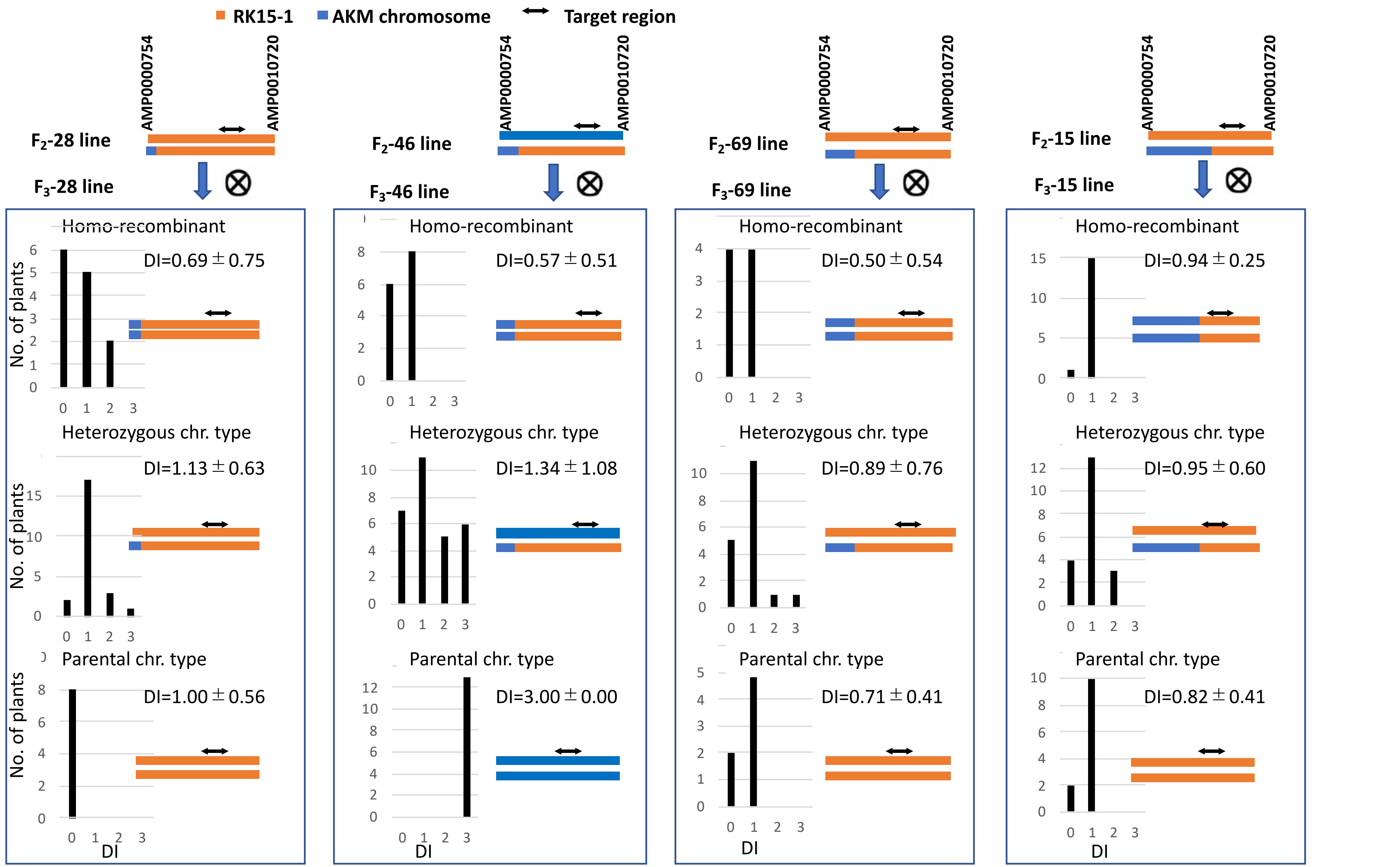

Supplemental Fig. 7. Relationship between YR phenotypes and recombination site and genotypes in the *ForRs1* region. *F. oxysporum* f.s. *raphani* inoculation data and chromosome segregation in the F_3 populations derived from self-pollination of the F_2 plants having parental and recombinant homologous chromosomes in the *ForRs1* locus. Inoculation data (No. of plants per DI class) and chromosome segregation of the subsequent F_3 progenies were indicated on the left and right side of each rectangle, respectively.

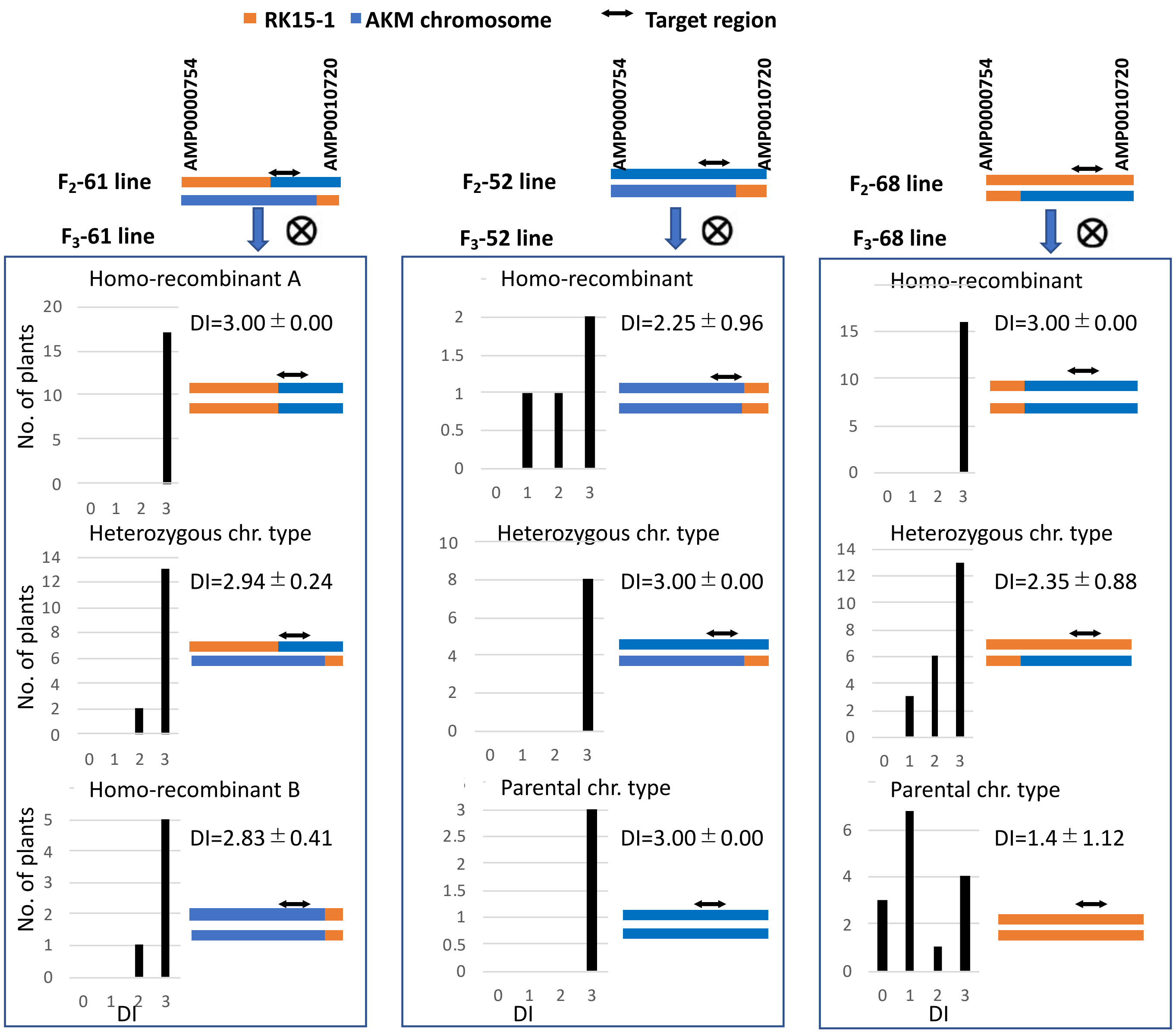

Supplemental Fig. 7 (cont.).

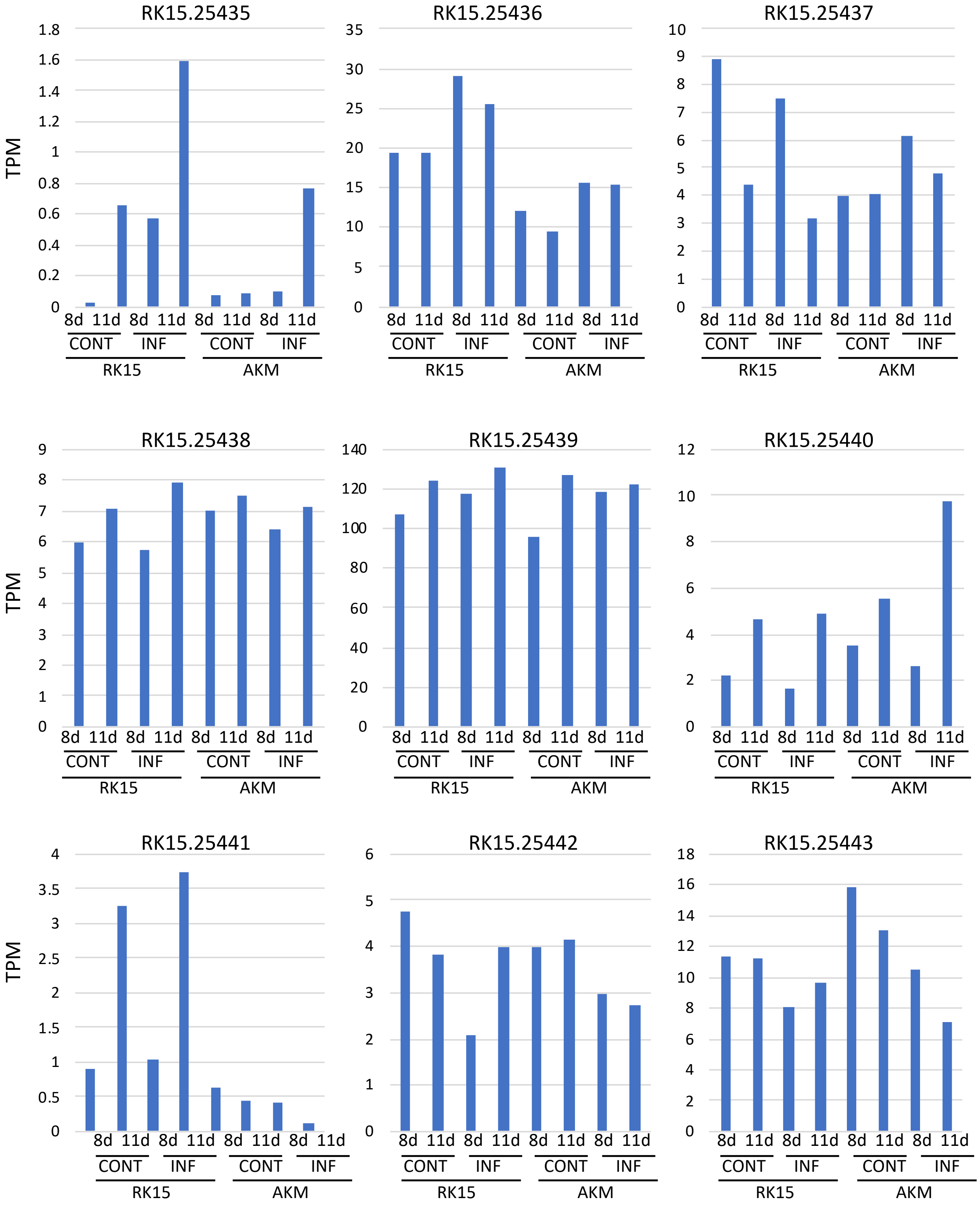

Supplemental Fig. 8. Transcripts per million (TPM) values obtained for 16 candidate genes in the RNA-seq analysis using seedlings grown under healthy (CONT) and diseased (INF) conditions for 8 and 11 days.

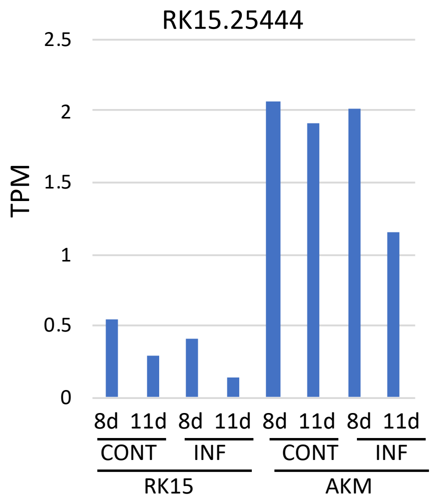
RK15.25444
TPM
2.5
2
1.5
1
0.5
0
8d 11d 8d 11d 8d 11d 8d 11d
CONT INF CONT INF
RK15 AKM

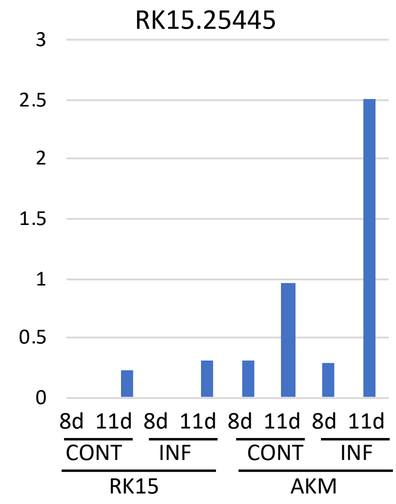
RK15.25445
3
2.5
2
1.5
1
0.5
0
8d 11d 8d 11d 8d 11d 8d 11d
CONT INF CONT INF
RK15 AKM

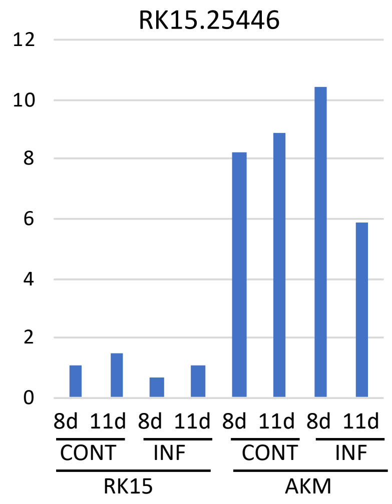
RK15.25446
12
10
8
6
4
2
0
8d 11d 8d 11d 8d 11d 8d 11d
CONT INF CONT INF
RK15 AKM

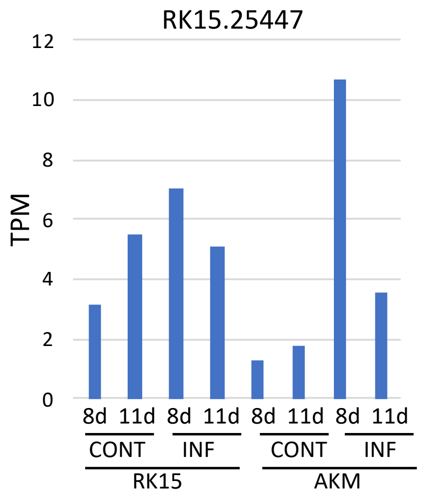
RK15.25447
TPM
12
10
8
6
4
2
0
8d 11d 8d 11d 8d 11d 8d 11d
CONT INF CONT INF
RK15 AKM

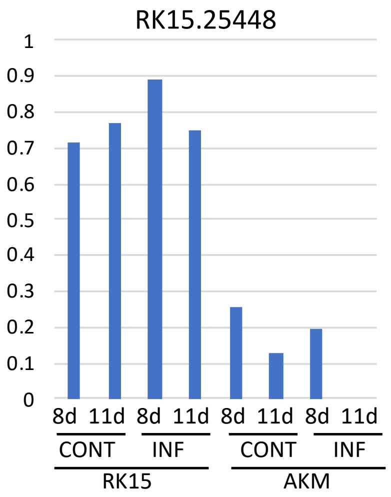
RK15.25448
1
0.9
0.8
0.7
0.6
0.5
0.4
0.3
0.2
0.1
0
8d 11d 8d 11d 8d 11d 8d 11d
CONT INF CONT INF
RK15 AKM

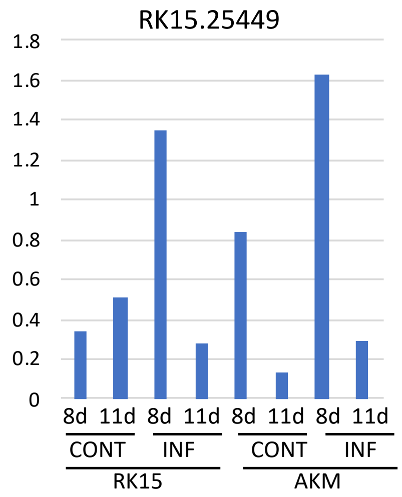
RK15.25449
1.8
1.6
1.4
1.2
1
0.8
0.6
0.4
0.2
0
8d 11d 8d 11d 8d 11d 8d 11d
CONT INF CONT INF
RK15 AKM

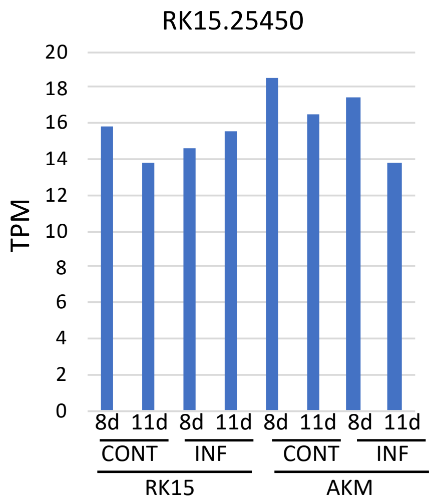
RK15.25450
TPM
20
18
16
14
12
10
8
6
4
2
0
8d 11d 8d 11d 8d 11d 8d 11d
CONT INF CONT INF
RK15 AKM

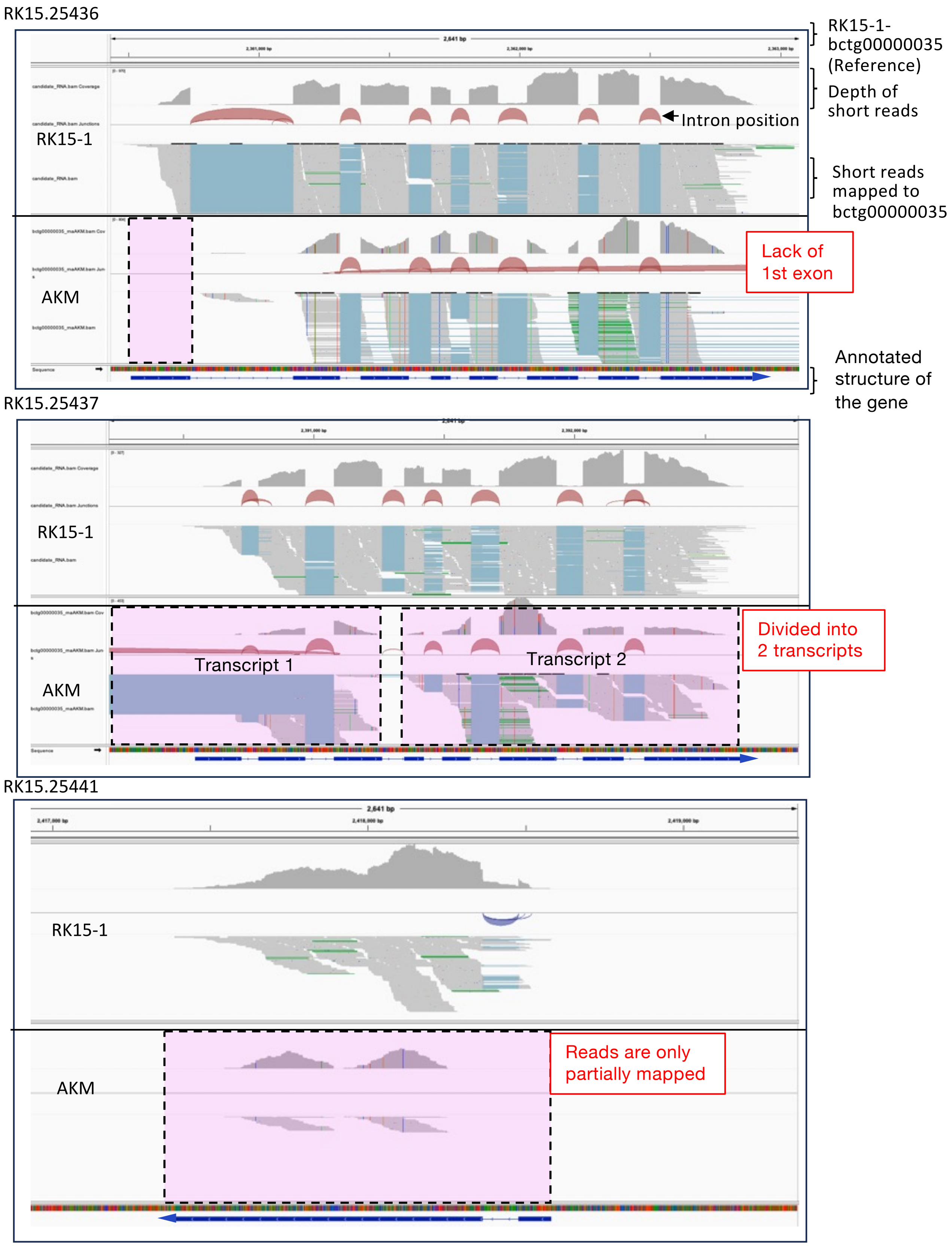

Supplemental Fig. 9. Transcription profiles observed in RK15-1 and AKM on the 3 genes (RK15.25436, RK15.25437, and RK15.25441). IGV views at the RK15-1 genome regions corresponding to the 3 genes are shown. RNA-seq data from different sampling points were pooled as one sample for each RK15-1 and AKM (see more details in Materials and Methods section). In each panel, RNA-seq reads mapped to the RK15-1 nanopore contig (bctg00000035) are shown in upper part, while RNA-seq reads from AKM are shown in lower part. At the bottom of each panel, the structure of the gene is indicated. Abnormal pattern of splicing and/or transcription initiation/termination of the 3 genes were observed in AKM.

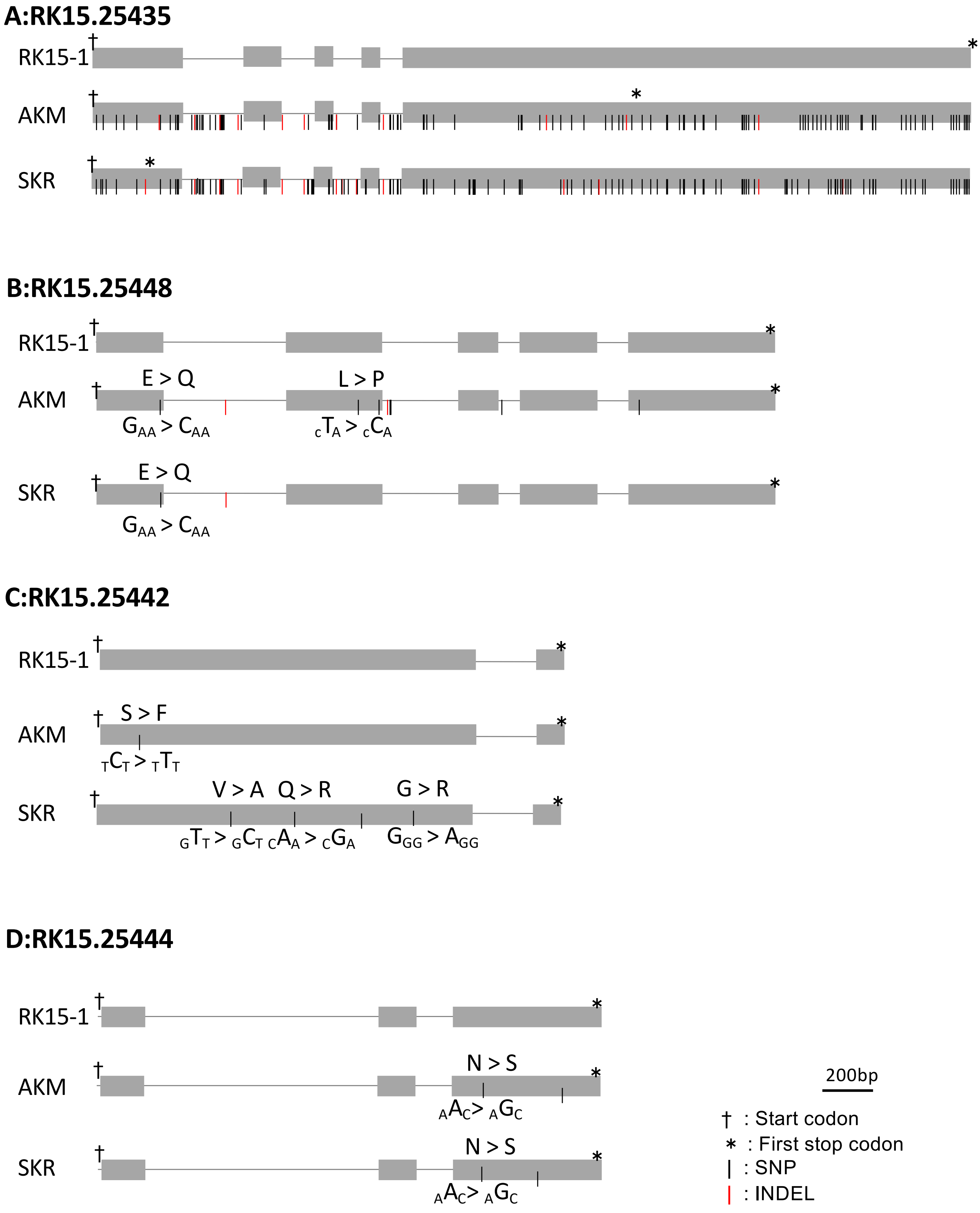

Supplemental Fig. 10. Schematic comparison of the four candidate genes among RK15-1, AKM, and SKR (Okute-Sakurajima). Boxes and horizontal lines indicate exons and introns, respectively.

```
PSKR1         1 ---------------------MRVHRFCVIVIFLTELLCFFYSSESQTTSRCHPHDLEAL 39
RK15.25438    1 ---------------------MRVWVVVIVLTELLLCFLWAWASESQTLLTCHPRDLEAL 39
RFO2          1 MTNEGRFKAKGFVRTSSTTRPIQALSFHMIGILLQCVLFISVLSIAVSEALCNSQDRESL 60

PSKR1        40 RDFIAHLEPKPDGWINSSSSTDCCNWTGITCNS-NNTGRVIRLELGNKKLSGKLSESLGK 98
RK15.25438   40 RDFISNLKPKPDDWS---IKDDCCNWTGVTCNNKNNSSRVTKLELGNKKLSGKLSESLGS 96
RFO2         61 LWFSGNVSSSVSPLN-WNPSIDCCSWEGITCDD-SPDSHITAISLPFRALYGKLPLSVLR 118

PSKR1        99 LDEIRVLNLSRNFIKDSIP---------LSIFNLKN----------------------LQ 127
RK15.25438   97 LDQITVLNLSRNFIRDSIP---------LSVFNLTT----------------------LQ 125
RFO2        119 LHHLSQLNLSHNRLSGHLPSGFLSALDQLKVLDLSYNSLDGELPVEQTFRNGSNRCFPIR 178

PSKR1       128 TLDLSSNDLSGGIP-TSINLP---ALQSFDLSSNKFNGSLPSHICHNSTQIRVVKLAVNY 183
RK15.25438  126 TLDLSSNDLSGEIPPNTVNLP---SLQSLDLSSNKLNGSLPSHLCRGSPRIRVIKLAVNY 182
RFO2        179 IVDLSSNFLQGEILPSSIFMQGTFDLISFNVSKNSFTGSIPSFMCKSSPQLSKLDFSYND 238

PSKR1       184 FAGNFTSGFGKCVLLEHLCLGMNDLTGNIPEDLFHLKRLNLLGIQENRLSGSLSREIRNL 243
RK15.25438  183 FAGDFPPGFGDCLLLEHLYLGMNNLTGNIPRDLFRLQRLNLLGVRENSLSGPLSPEIGSL 242
RFO2        239 FTGNIPQGLGRCLKLSVLQAGFNNISGEIPSDIYNLSELEQLFLPVNHLSGKINDDITHL 298

PSKR1       244 SSLVRLDVSWNLFSGEIPDVFDELPQLKFFLGQTNGFIGGIPKSLANSPSLNLLNLRNNS 303
RK15.25438  243 SGLVRLDVSSNRFSGEIPDVFGEMTRLKELLARSNRFTGGIPKSLSNSPTLNLVNLRNNS 302
RFO2        299 TKLKSLELYSNHLGGEIPMDIGQLSRLQSLQLHINNITGTVPPSLANCTNLVKLNLRLNR 358

PSKR1       304 LSGRL-MLNCTAMIALNSLDLGTNRFNGRLPENLPDCKRLKNVNLARNTFHGQVPESFKN 362
RK15.25438  303 LTGPL-QLNCTAMVGLNSLDLGTNRFNGRLPENLPVCKRLKNLNLARNSFHGQVPESFKD 361
RFO2        359 LEGTLSELDFSRFQSLSILDLGNNSFSGDFPWRVHSCKSLSAMRFASNKLTGQISPHVLE 418

PSKR1       363 FESLSYFSLSNSSLANISSALGILQHCKNLTTLVLTLNFHGEALPDDSS----LHFEKLK 418
RK15.25438  362 FQSLSFFSLSNSSLSNISSALRILQSCKNLTSLVITLNFHGEALPDGDSSL--LHFEKLK 419
RFO2        419 LESLSILSLSDNKLMNITGALGILQGCRNLSTLLIGKNFYNETFPSDKDLISSDGFPNLQ 478

PSKR1       419 VLVVANCRLTGSMPRWLSSSNELQLLDLSWNRLTGAIPSWIGDFKALFYLDLSNNSFTGE 478
RK15.25438  420 VLVIANCKLTGSMPRWLTSSNDLQLLDLSWNRLTGAIPSWIGDFKDLFYLDLSNNSFTQE 479
RFO2        479 IFASGGSGLRGEIPAWLIKLKSLAVIDLSHNQLVGSIPGWLGTFPHLFYIDLSENLLSGE 538

PSKR1       479 IPKSLTKLESLTSRNISVNEPS--PDFPFFMKRNESARALQYNQIFGFPPTIELGHNNLS 536
RK15.25438  480 IPKSLTKLLSLTSRNISLDEPS--PDFPFFMKRNESARGLQYNQIVGFPPTIELGHNNLS 537
RFO2        539 LPKDLFQLKALMSQKAYDATERNYLKLPVFVSPNNVTTHQQYNQLFSLPPGIYIRRNNLK 598

PSKR1       537 GPIWEEFGNLKKLHVFDLKWNALSGSIPSSLSGMTSLEALDLSNNRLSGSIPVSLQQLSF 596
RK15.25438  538 GPIWEEFGNLKKIHVFDLKWNSLSGNIPSSLSGMTNLEVLDLSNNHLSGSIPSSLQKLTF 597
RFO2        599 GSIPIEVGQLKVLHVLELSHNYLSGIIPHELSKLTSLERLDLSNNHLSGRIPWSLTSLHY 658

PSKR1       597 LSKFSVAYNNLSGVIPSGGQFQTFPNSSFESN-HLCGEHRFPCSE----GTESALIK-RS 650
RK15.25438  598 LSKFSVASNNLSGRIPSGGQFQTFPNSSFEFN-DLCGEHRLPCSEDAVNGTTTLMIHSRR 656
RFO2        659 MSYFNVVNNSLDGPIPTGSQFDTFPQANFKGNPLLCGGILLTSCKASTKLPATTTNKADT 718

PSKR1       651 RRSRGGDIGMAIGIAFGSVFLLTLLSLIVLRARRRSGEVDPEIEESESMNRKELGEIGSK 710
RK15.25438  657 RRSREVEIGMAAGIASGSVFLLTILALEVFSPN-----------------------ILEK 693
RFO2        719 EDEEELKFIFILGVATG--FFVSYCFYWCFFAR--------------------------- 749

PSKR1       711 LVVLFQSNDKELSYDDLLDSTNSFDQANIIGCGGFGMVYKATLPDGKKVAIKKLSGDCGQ 770
RK15.25438  694 LMKLFKIH---------------------------------------------------- 701
RFO2        750 -LDAFISK---------------------------------------------------- 756

PSKR1       771 IEREFEAEVETLSRAQHPNLVLLRGFCFYKNDRLLIYSYMENGSLDYWLHERNDGPALLK 830
RK15.25438  701 ------------------------------------------------------------ 701
RFO2        756 ------------------------------------------------------------ 756

PSKR1       831 WKTRLRIAQGAAKGLLYLHEGCDPHILHRDIKSSNILLDENFNSHLADFGLARLMSPYET 890
RK15.25438  701 ------------------------------------------------------------ 701
RFO2        756 ------------------------------------------------------------ 756

PSKR1       891 HVSTDLVGTLGYIPPEYGQASVATYKGDVYSFGVVLLELLTDKRPVDMCKPKGCRDLISW 950
RK15.25438  701 ------------------------------------------------------------ 701
RFO2        756 ------------------------------------------------------------ 756

PSKR1       951 VVKMKHESRASEVFDPLIYSKENDKEMFRVLEIACLCLSENPKQRPTTQQLVSWLDDV   1008
RK15.25438  701 ----------------------------------------------------------   701
RFO2        756 ----------------------------------------------------------   756
```

Supplemental Fig. 11. Alignment of amino acid sequences of PSKR1, RK15.25438 and RFO2.

1st Amino acid sequence: RK15-1. 25435 Sequence size: 951
2nd Amino acid sequence: AKM. 25435 Sequence size: 927

```
  1' MEGKLFVGQY LILVISLLGQ LHGYISCIEK EMVALLDLKK YTIASNESNQ FLTDWTNLTK
     ********** ***** **** ********** *.******** *.******** *******.**
  1" MEGKLFVGQY LILVILLLGQ LHGYISCIEK ERVALLDLKK YSIASNESNQ FLTDWTNVTK

 61' SDCCQWEKVK CDRASGRVIR LSIGW--AHH RESSLNLSLL HPFEEIQILD LSWSGFSGLF
     ********** ********** *****  **. ********** ********** *** **..**
 61" SDCCQWEKVK CDRASGRVIR LSIGWARAHY RESSLNLSLL HPFEEIQILD LSWLGFGSLF

119' DDVEGYKSLR RLRYLEILDL SVNAFNNSIF PFINAATSLI TLLLPLNNME GLFPAKEFKD
     ********** ********** ********** ********** ********** *****.****
121" DDVEGYKSLR RLRYLEILDL SVNAFNNSIF PFINAATSLI TLLLPLNNME GLFPANEFKD

179' LTNLELLDLS ENRFNRSIPL GDLPALMNLK ALDLHGNEFS TSVELQGICE LKNLEELNLS
     ********** ********** ********** ********** ********** **********
181" LTNLELLDLS ENRFNRSIPL GDLPALMNLK ALDLHGNEFS TSVELQGICE LKNLEELNLS

239' QNKLVGQFPL CLPSFNGLRV LDLSSNQLNE RLPSAIRNLE SLEYMSLSNN SFKGSFSLGL
     ********** ***. ***** ********** ********** ********** **** *****
241" QNKLVGQFPL CLPNSNGLRV LDLSSNQLNE RLPSAIRNLE SLEYMSLSNN SFKGFFSLGL

299' LANLSKLRVF RLDSKGNSLQ VKSGSAWKPK FQLNVIALPS CNLKKVPHFL LHQKDLRHVD
     ********** ********** ********** ********** ********** **********
301" LANLSKLRVF RLDSKGNSLQ VKSGSAWKPK FQLNVIALPS CNLKKVPHFL LHQKDLRHVD

359' LSDNNISGTF PYWLLVNNTK LENLHLQNNF FTGFQLPEIS HHNLHWLDLS VNNLTGLLPE
     ********.* * ******** ********** *******..* ********** .*********
361" LSDNNISGSF PSWLLVNNTK LENLHLQNNF FTGFQLPXXS HHNLHWLDLS INNLTGLLPE

419' SIGWILPSLS YMNISENGFE GNLPSSLGNM GSVEYMDLSR NSFHGKLPRA LVEGCYSLVI
     *******.** ********** ********** **.******* ********** **********
421" SIGWILPNLS YMNISENGFE GNLPSSLGNM GSIEYMDLSR NSFHGKLPRA LVEGCYSLVI

479' LKVSCNKLSG EIFQESVSFT GIYALFLDNN QFTGEIGHGL RRLINLLLLD ISNNNLTGVI
     ****.***** *.*****.** ***** .  .     *  . .    .   *     .  .
481" LKVSYNKLSG EVFQESVNFT GIYALCFWIT ISLQEKLDMV YGGTCYCLTF QMAIPVLFQA

539' PSWIGELPTL FSLLLSNNSL EGEIPISLVS MSSIRLLDLS SNSLSGSIPP HVTSGTSVVF
         ..     ...  .. .. .  .  *  . . .      .     .       ..
541" GLANSQRYFR YCFQTTHWKV KYLFLWSAYP VLGYLTSPQT LYLGPYLQML LLELQCYCKT

599' LLKDNNLSGV IPNTLLLNVS LLDLRNNRFS GNIPEFISNQ RASFLLLRGN NLTGQIPSQL
     .. .. . .      * *          ..   ... ...     ....  *   .   . .  .
601" IIYQGLFQTH CCMFLYLIEI TDFQGISQSL SATKGLVFFF CVGIIQGRFL ASYVRQAFTF

659' CALTSIRLLD LANNRLSGSI PSCLSNISSD LRKVYTPVYD FGIDSQELSQ LSYLDRDSIS
       * .* ...   .      *  .     *     . . . .   .. .. ..    .    .  .
661" WILLTIDVVQ YLHVYKQYII FEKSVYASLF WHFTRIVTTF LSGQGLHFHT RCRYLFQIYA

719' TRDVGTYFKS LLILDQFTTD GYDVGTQTKI EFATKGRFDA YMGSNLGYLI GMDLSENELS
                    ..  .  .              . .     ....       .. . . ..
721" YTRSVYKRWI CSHPNENRIC NKRQIRLHGQ PRIFDWNGPL RKAERDFSRA WRSYGVSCFK

779' GEIPAELGGL KEFHALNLSH NSLSGAIPES FSGLKNMESL DLSYNRVQGP IPPQLAELSS
     .  .. .    .     . .. .. *   ...  *. .. ..  . . .       .   .  ..
781" SFSQQFFRCY TRKLFRSEEY GKPSFLQQTT RSDPTTTSRA EQPRSLQCII QQLIRSYSTG

839' LAVFNVSYNN LSGVIPLGRQ FNTFETKSYL GNPLLCGKPT NISCESNNFQ EADDEVEADE
      .*   .  .  . .   ...   . . ..   ... . ..    .  **      .... .
841" KTVHLDKLLR SPFLWGTNQH KLPEQRSRSR SGSGIHNRYR IFLLESCCSL CDHTHWNICI

899' STIDTESFYW SLVAAYATTL IGIFASF
       .  .      ..  .     *. ..
901" FLFRFSLEQI LVLHRCFHRQ IEELVVV
```

Supplemental Fig. 12. Alignment of amino acid sequences in the RK15.25438 genes of RK15-1 and AKM.
